# *Cuscuta campestris* fine-tunes gene expression during haustoriogenesis as an adaptation to different hosts

**DOI:** 10.1093/plphys/kiad505

**Published:** 2023-09-14

**Authors:** Thomas Bawin, Alena Didriksen, Corine Faehn, Stian Olsen, Iben Sørensen, Jocelyn K C Rose, Kirsten Krause

**Affiliations:** Department of Arctic and Marine Biology, UiT The Arctic University of Norway, Tromsø 9019, Norway; Department of Arctic and Marine Biology, UiT The Arctic University of Norway, Tromsø 9019, Norway; Department of Arctic and Marine Biology, UiT The Arctic University of Norway, Tromsø 9019, Norway; Department of Arctic and Marine Biology, UiT The Arctic University of Norway, Tromsø 9019, Norway; Plant Biology Section, School of Integrative Plant Science, Cornell University, Ithaca, NY 14853, USA; Plant Biology Section, School of Integrative Plant Science, Cornell University, Ithaca, NY 14853, USA; Department of Arctic and Marine Biology, UiT The Arctic University of Norway, Tromsø 9019, Norway

## Abstract

The *Cuscuta* genus comprises obligate parasitic plants that have an unusually wide host range. Whether *Cuscuta* uses different infection strategies for different hosts or whether the infection strategy is mechanistically and enzymatically conserved remains unknown. To address this, we investigated molecular events during the interaction between field dodder (*Cuscuta campestris*) and two host species of the *Solanum* genus that are known to react differently to parasitic infection. We found that host gene induction, particularly of cell wall fortifying genes, coincided with a differential induction of genes for cell wall degradation in the parasite in the cultivated tomato (*Solanum lycopersicum*) but not in a wild relative (*Solanum pennellii*). This indicates that the parasite can adjust its gene expression in response to its host. This idea was supported by the increased expression of *C. campestris* genes encoding an endo-β-1,4-mannanase in response to exposure of the parasite to purified mono- and polysaccharides in a host-independent infection system. Our results suggest multiple key roles of the host cell wall in determining the outcome of an infection attempt.

## Teaser

The gene expression of cell wall-degrading enzymes in the parasitic plant *Cuscuta campestris* is regulated in response to purified polymers and the cell wall composition of a given host.

## Introduction

Parasitic behaviors in angiosperms evolved independently at least twelve times (Nickrent 2020) and involve associations with a broad range of plant hosts. Examples include species from the *Cuscuta, Orobanche*, and *Striga* genera, which can cause serious damage to important crops because they are difficult to eradicate due to their tight physical connections and metabolomic overlap with their hosts (Aly 2007; Parker 2012; Vurro et al. 2017). A common feature of parasitic plants is the presence of a specialized organ known as the haustorium, which penetrates the host tissue and enables a parasite to sequester water, inorganic salts, and organic compounds, creating a nutrient sink (Westwood et al. 2010; Kim and Westwood 2015). Given their agricultural importance, there is interest in determining the mechanisms by which parasitic plants overcome host defenses, as well as the molecular pre- and postattachment events in susceptible and resistant plant species (Fishman and Shirasu 2021; Jhu and Sinha 2022). Such information may lead to the identification of specific targets in the parasites or hosts that can be used to mitigate infection and support successful and cost-effective pest management.

To this end, a useful experimental model is *Cuscuta* sp. (dodders), a genus of obligate parasites that are the only parasitic member of the Convolvulaceae family, with about 200 species and a worldwide distribution (García et al. 2014; Costea et al. 2015). *Cuscuta* species appear as thread-like shoots that grow in a counter-clockwise motion around the aerial parts of the hosts and develop lateral haustoria at the interface. Firm attachment of the parasite to the host is first promoted by sticky substances (Vaughn 2002) and coincides with a swelling of the *Cuscuta* stem and a reshaping of the epidermal cells into club-shaped cells. Haustoria then develop invasive structures and specialized “feeding hyphae” at the tip and the sides connect the parasite to the vascular cells of the host (Vaughn 2003; Vaughn 2006; Shimizu and Aoki 2019), a process that is influenced by host molecular mechanisms (Liu et al. 2020; Narukawa et al. 2021).

Plants have evolved to activate immune responses and promote healing because they are constantly attacked by other organisms. The best-described example of active resistance to dodders is the strong reaction induced by giant dodder (*Cuscuta reflexa*) in cultivated tomato (*Solanum lycopersicum*) that prevents the parasite from growing and propagating. Upon attachment, tomato epidermal cells expand and die in a way that is reminiscent of a hypersensitive response, while a suberin-like barrier is formed in the underlying tissues to prevent penetration by haustoria (Kaiser et al. 2015; Albert et al. 2021). A cell surface leucine-rich repeat protein, the *Cuscuta Receptor 1 (CuRe1)*, was shown to recognize a glycine-rich protein in the *C. reflexa* cell wall and to trigger classical defense-related responses, including a reactive oxygen species burst and the production of the phytohormone ethylene (Hegenauer et al. 2016, 2020). Furthermore, several loci mapping to different chromosomes in a population of cultivated tomato individuals that were introgressed with genome fragments of the closely related and fully susceptible *Solanum pennellii* revealed the presence of other genes associated with tomato resistance to *C. reflexa* (Krause et al. 2018). Recently, a cytosolic receptor encoded by the *Cuscuta R-gene for Lignin-based Resistance 1* (*CuRLR1*) was suggested to confer resistance against field dodder (*Cuscuta campestris*) in a few *S. lycopersicum* Heinz cultivars via local lignification upon attachment and prevention of haustorial penetration (Jhu et al. 2022). Other potential receptors in the cultivated tomato involved in perceiving signals from *Cuscuta* parasites include *CuRe1*-like, pathogenesis-related, and nucleotide-binding leucine-rich repeat proteins (Hegenauer et al. 2016; Jhu et al. 2021a).

In addition to lignin, plant cell walls consist of diverse complex polysaccharides including cellulose, hemicelluloses, and pectins that interact to form a complex matrix. Cell wall composition and architecture vary among cells, tissues, and species and continuously change during growth and development and in response to stress (Gigli-Bisceglia et al. 2020). The cell wall acts as a physical barrier that must be overcome by parasites. As a result, pathogens have evolved an arsenal of cell wall-degrading enzymes, which are key virulence factors. The diversity of these enzymes reflects the structural and dynamic complexity of the plant cell wall and the lifestyle and host adaptation of pathogens (Bellincampi et al. 2014; Kubicek et al. 2014; van der Does and Rep 2017). Cell wall fragments that are released and accumulate upon mechanical injuries and/or enzymatic degradation can serve as damage-associated molecular patterns (DAMPs) and cause hypersensitivity and death of individual cells (Bacete et al. 2018; Pontiggia et al. 2020). Evidence shows that cell wall-related enzymes play a central role in the interaction between parasitic plants, including *Cuscuta* species, and their hosts. Pectin, which binds cells together and influences cell wall porosity and thickness, is a key target for degradation during parasitic infection because its alteration allows for loosening of the host tissues and penetration of intrusive cells (Kokla and Melnyk 2018; Shimizu and Aoki 2019; Yokoyama et al. 2021). Highly regulated genes coding for xyloglucan endotransglucosylase/hydrolase enzymes in dodders were demonstrated to promote haustorial growth into the host and were suggested to participate in forming vascular connections by hyphae (Olsen et al. 2016a; Olsen and Krause 2017). However, little is known about the influence of the host on the expression of *Cuscuta* genes encoding cell wall-related enzymes and their action as virulence factors and about the effects of cell wall degradation products on host immunity and their relative importance compared to other defense components.

In the present study, we investigated gene-for-gene relationships during the parasitization process using *S. lycopersicum* cv. M82 and its wild relative *S. pennellii* (LA0716) as closely related hosts. We emphasize the influence of host cell wall composition in the arms race with *Cuscuta* parasites and stress the strategic importance of the latter to win the battle at the cell wall interface.

## Results

### A temporal resolution strategy for the simultaneous analysis of host and parasite transcriptomes at the infection site

**Figure 1.**
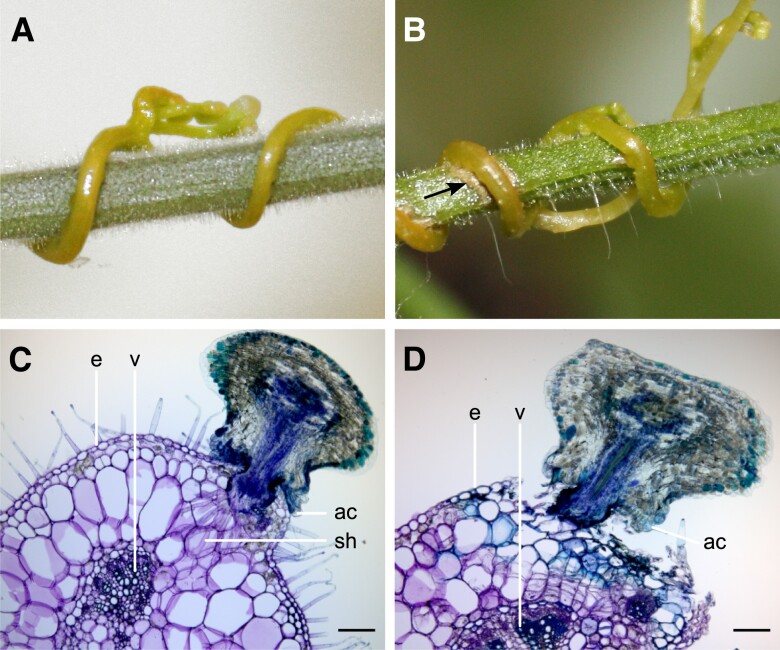
*Cuscuta campestris* parasitizing *Solanum pennellii* (LA0716) versus *S. lycopersicum* cv. M82. **A)** Parasitism does not induce a visible reaction in *S. pennellii*, whereas **B)** a necrotic lesion is formed at the penetration site (arrow) in *S. lycopersicum*. **C)** Cross section of a *C. campestris* haustorium that is attached to an *S. pennellii* petiole. The holdfast contributes to the adhesion of the parasite to the host via adhesive cells, while intrusive searching hyphae develop from haustorial digitate cells. **D)** Cross section of a *C. campestris* haustorium that is attached to an *S. lycopersicum* petiole. Cells stained blue with TBO underneath the attachment ring, presumably due to the accumulation of phenolic compounds, indicate a defense response within the partially peeled-off wound tissue that restrains penetration by the haustorium, which is similar to the response previously described with *C. reflexa* on the same cultivar (Krause et al. 2018). All scale bars are 200 *μ*m. ac = adhesive cell, e = petiole epidermis, sh = searching hyphae, v = petiole vascular bundle, TBO = Toluidine blue O.

A challenge in characterizing molecular processes at the host-parasite interface during infection is the close connection between the two. We revisited the *Cuscuta*-tomato pathosystem with *S. pennellii* (LA0716) and *S. lycopersicum* cv. M82 because they are two closely related representatives of the *Solanum* genus that clearly differ in their responses to dodder attack. Both species allow successful propagation of *C. campestris*; however, the parasite induces a defense reaction only in *S. lycopersicum*, which includes a hypersensitive-like response and changes in cell wall composition as visualized by color alterations after staining cross-sections of the infection site with the polychromatic stain Toluidine Blue O (TBO) (Fig. 1). Infection sites containing parasite and host tissues were sampled individually and assigned to one of the previously defined stages of haustorium development (swelling, attachment, and penetration) using the established marker gene sets (Supplemental Fig. S1 and Data sets S1 to S2) (Bawin et al. 2022). Mapping of transcriptome sequencing reads to a chimeric assembly of the *C. campestris* and *S. lycopersicum* genomes showed that 90.5% to 96.5% of read pairs (fragments) aligned uniquely and that 91.6% to 94.2% of these were assigned to the total gene models (Supplemental Fig. S2 and Tables S1 and S2).

### *C. campestris* gene expression changes in response to host exposure

**Figure 2.**
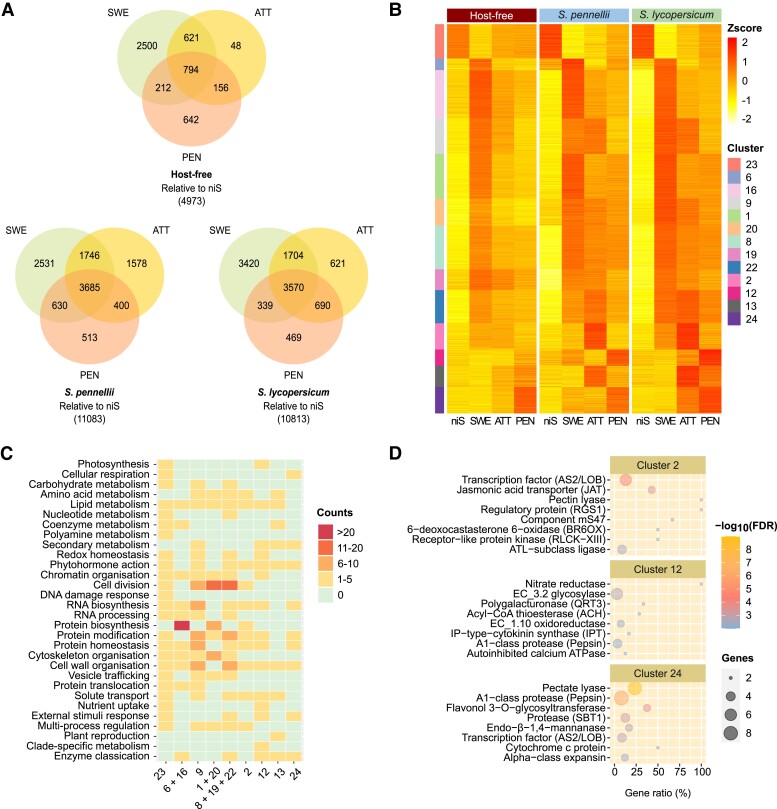
Gene expression dynamics in *Cuscuta campestris*. **A)** Representation of Venn intersections relative to niS. Numbers represent differentially expressed genes. Only genes that showed a FDR-corrected *P*-value ≤ 0.05 and a |log_2_(fold change)| ≥ 1.5 were retained. **B)** Selected soft clusters of differentially expressed genes as a function of haustorial development stages. Values are average z-scores obtained from TPM counts. A z-score value is positive (negative) if the gene expression in a sample type is larger (smaller) than the overall mean expression. Genes were sorted by cluster then decreasing membership value. Only genes with a membership ≥ 0.7 were retained. **C)** Schematic representation of MapMan4 v.4.0 enrichment in functional categories as a function of clusters. Counts refer to the number of bins that are significantly enriched with genes inside each top-level category. Redundant clusters (with an overall similar pattern of expression) were grouped together as supported by a correlation test (see Supplemental Fig. S3B). **D)** Top enriched functional terms in clusters 2, 12, and 24. Gene ratio refers to the number of genes found in a cluster with a term relative to the number of genes in the transcriptome with that term. HF = host-free, SP = *Solanum pennellii*, SL = *S. lycopersicum*, niS = noninfective stem, SWE = swelling stage, ATT = attaching stage, PEN = penetrating stage, FDR = false discovery rate, TPM = transcripts per kilobase million.

To characterize host-influenced gene expression in the parasite, we compared infection sets for both tomato species with each other and with an earlier host-free experiment (Bawin et al. 2022). Differential expression analysis involving comparisons between infective with noninfective *C. campestris* tissues revealed that roughly twice as many genes (11,083 and 10,813 vs. 4,973) were upregulated or downregulated in the presence of a host (Fig. 2A). Transcriptional dynamics during the transition from noninfective to infective structures were then examined using a soft-clustering approach. A total of 8,774 genes that were differentially expressed in at least one of the three interaction systems were compiled, and genes with similar expression profiles were grouped in 25 clusters (Supplemental Figs. S3 to S4 and Data Set 3). Eight of these clusters involved genes whose upregulation marked the onset of haustorium development and for which higher expression was often sustained in subsequent stages (Fig. 2B). These clusters bore a clear signature of growth and attachment processes (Fig. 2C). Four clusters further comprised of genes that were specifically upregulated in the later stages (Fig. 2B). Among these, clusters 2 and 12 contained genes that were upregulated during attachment and penetration, respectively, in the presence of either of the two hosts. The most significant functional enrichment in cluster 2 concerned ASYMMETRIC LEAVES2/LATERAL ORGAN BOUNDARIES (AS2/LOB) transcription factors (Fig. 2D), while cluster 12 was enriched in categories that included “phytohormone action”, “solute transport”, and “enzyme classification” (Fig. 2C). The latter included several polygalacturonases and glucan endo-1,3-β-glucosidases. Genes in clusters 13 and 24 were more strongly expressed in *S. lycopersicum* than in the two other interaction systems (Fig. 2B). Cluster 13 included two 1-aminocyclopropane-1-carboxylic acid (ACC) oxidase (*ACO*) genes, associated with ethylene biosynthesis, which showed a dramatic increase in expression. Cluster 24 contained numerous lytic enzymes, such as peptidases, proteases, pectate lyases, and endo-β-1,4-mannanases (Fig. 2D). Several transcription factor families were also represented, including the AS2/LOB gene *CcLBD25* (Cc019141), which was previously linked to pectin degradation and the development of searching hyphae (Jhu et al. 2021b).

### The host has a strong influence on cell wall-related gene expression

**Figure 3.**
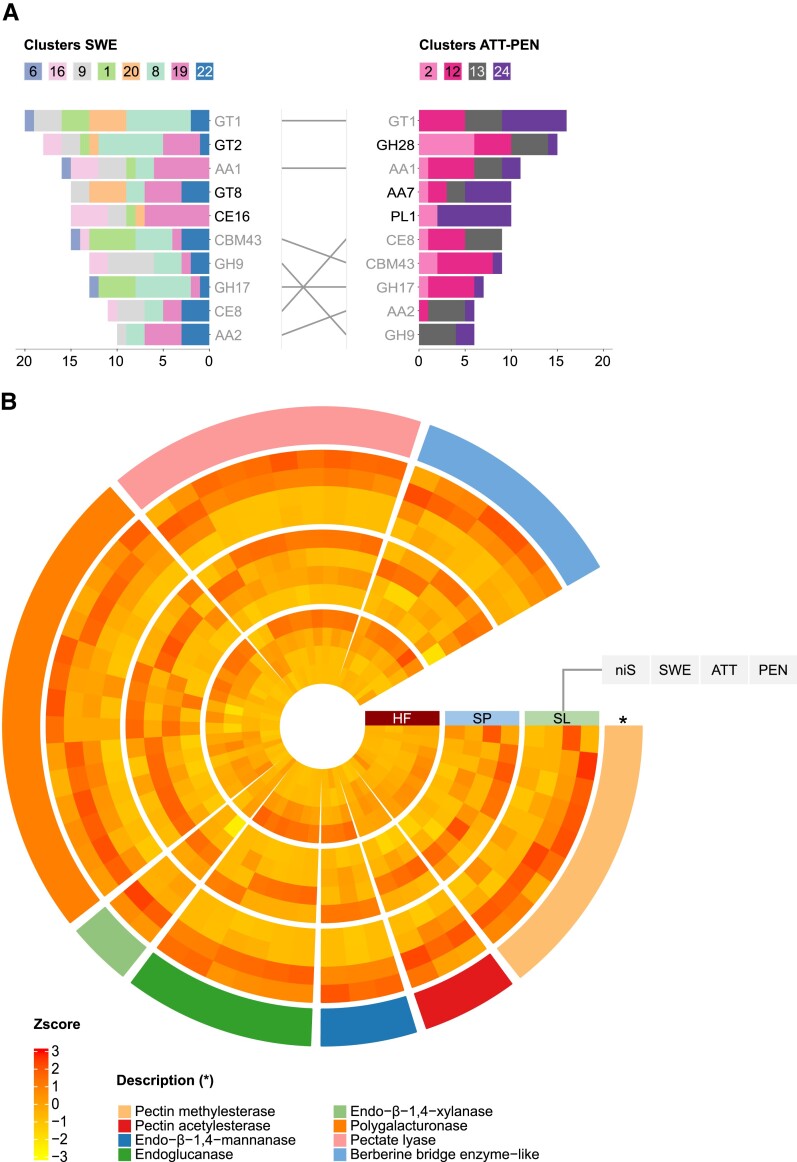
Putative CAZyme genes in *Cuscuta campestris*. **A)** Number of CAZyme motifs per family contained in clustered accessions. The left panel shows motif numbers from genes that contributed to the onset of haustorium development (SWE). The right panel shows motif numbers from genes that showed specific upregulation during ATT-PEN. Only the 10 most represented CAZyme families are displayed on each side. Families that are common to both panels are shaded and linked. **B)** Expression pattern of genes assigned to selected CAZyme families, including cell wall-degrading enzymes, which showed specific upregulation during attachment and attempted penetration. Values are average z-scores obtained from TPM counts. A z-score value is positive (negative) if the gene expression in a sample type is larger (smaller) than the overall mean expression. Genes are sorted by CAZyme family (outermost layer). HF = host-free, SP = *S. pennellii*, SL = *S. lycopersicum*, niS = noninfective stem, SWE = swelling stage, ATT = attaching stage, PEN = penetrating stage, TPM = transcripts per kilobase million.

To gain deeper insight into the potential involvement of *C. campestris* cell wall-related genes during parasitism, we investigated the expression of genes encoding Carbohydrate-Active Enzymes (CAZymes) (http://www.cazy.org) in the different haustorial stages. From the clusters associated with the onset of haustorium development, 269 putative CAZyme genes (7.46% of 3,605 accessions) from 50 families were identified. The most represented families contained galacturonosyltransferases (from the Glycosyl Transferase (GT) family 8), cellulose synthase-like genes (GT2) that included a wide array of mannan synthases, and GDSL esterase/lipase genes (Carbohydrate Esterase (CE) family 16) (Fig. 3A). Of these, many had their expression level sustained in the later stages but were never specifically expressed, supporting a possible role in growth, morphogenesis, and cell wall patterning throughout haustoriogenesis. Clusters associated with attachment and attempted penetration of host tissues (ATT-PEN) contained 147 putative CAZyme genes (14.03% of 1,048 accessions) from 38 families. These included polygalacturonases (Glycosyl Hydrolase (GH) family 28), pectate lyases (Polysaccharide Lyase (PL) family 1), pectin methylesterases (PMEs/CE8), and pectin acetylesterases (PAEs/CE13), all of which were strongly induced in either haustoria infecting a living host compared to host-free haustoria or in haustoria infecting *S. lycopersicum* compared to the other two interaction systems (Figs. 3, A and B). Those pectin-related genes accounted for a quarter (25.17%) of all the identified CAZyme accessions for the two later stages, underpinning the previously recognized role of pectin remodeling and degradation in the host cell wall by the parasite. Similar evidence was obtained with endo-β-1,4-mannanases (GH5), endo-β-1,4-xylanases (GH10), and endo-β-1,4-glucanases (GH9) exhibiting differential expression between the interaction systems, further stressing the importance of the host (hemi)celluloses (Fig. 3B). In addition, differentially expressed genes coding for berberine bridge enzyme-like proteins (Auxiliary Activities (AA) family 7) were among the most represented in the later stages, with 7 of the 20 family members identified in the whole transcriptome by MapMan4 (Schwacke et al. 2019) being scattered across the four decisive clusters (Figs. 3, A and B). In contrast, none were identified among the genes associated with the onset of haustorium development, supporting the notion that these oxidases can act in conjunction with cell wall-degrading enzymes during the invasion of a host.

### The two *Solanum* hosts show profoundly different responses to a *Cuscuta* attack

**Figure 4.**
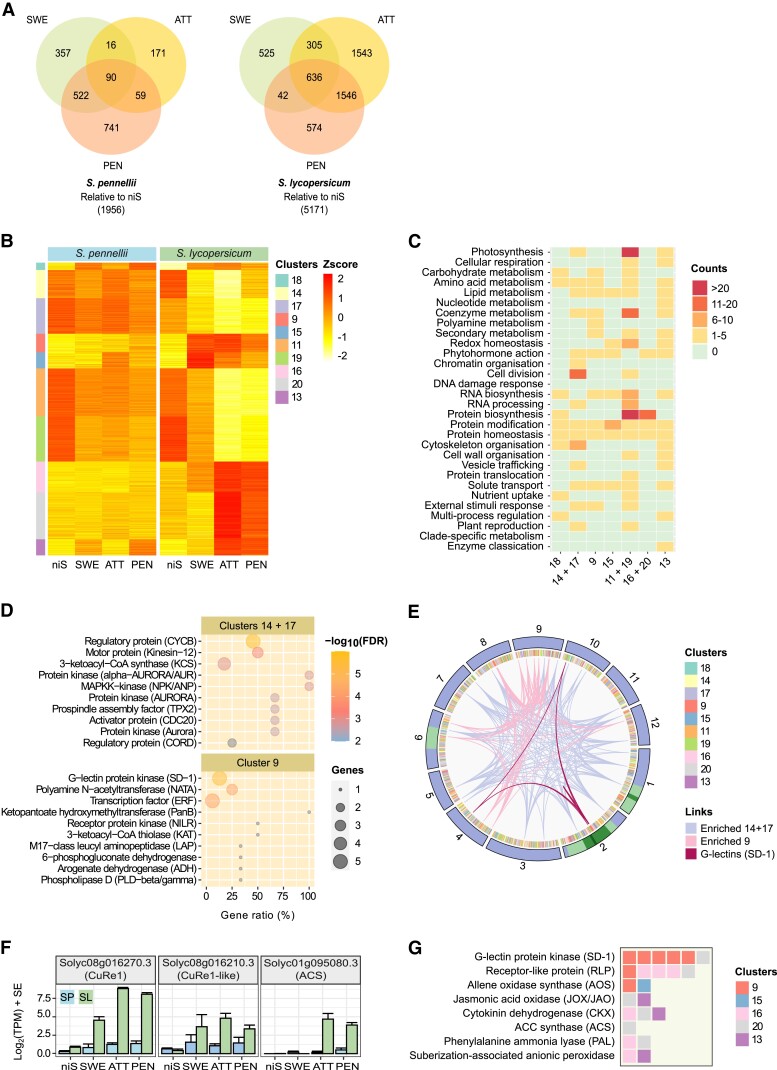
Gene expression dynamics in tomato hosts. **A)** Representation of Venn intersections relative to niS. Numbers represent differentially expressed genes. Only genes that have a FDR-corrected *P*-value ≤ 0.05 and a |log_2_(fold change)| ≥ 1.5 were retained. **B)** Selected soft clusters of differentially expressed genes as a function of haustorial development stages. Values are average z-scores obtained from TPM counts. A z-score value is positive (negative) if gene expression in a sample type is larger (smaller) than the overall mean expression. Genes are sorted by cluster then decreasing membership value. Only genes with a membership ≥ 0.7 were retained. **C)** Schematic representation of MapMan4 v.4.0 enrichment in functional categories as a function of clusters. Counts refer to the number of bins that are significantly enriched with genes inside each top-level category. Redundant clusters (with an overall similar pattern of expression) were grouped together as supported by a correlation test (see Supplemental Fig. S5B). **D)** Top enriched functional terms in combined clusters 14 and 17, and in cluster 9. Gene ratio refers to the number of genes found in a cluster with a term relative to the number of genes in the transcriptome with that term. **E)** Visualization of *Solanum lycopersicum* chromosomes (outer layer). The length of chromosome segments is proportional to the number of genes contained in the soft clusters selected in (B). Genes are sorted by chromosome coordinates. Green color refers to the position of introgressed regions that mediate full susceptibility of M82 to *Cuscuta* infection (Krause et al. 2018); a darker color indicates overlap between regions. Cluster assignment is displayed for each gene (inner layer). Lines connect genes from the enriched functional categories in (D). **F)** Expression profile of genes coding for two receptor-like proteins (*CuRe1* and *CuRe1*-like) and an ACC synthase. Values are average log-transformed TPM in sequenced samples. Error bars indicate standard error (SE) of the mean (three biological replicates). **G)** Cluster distribution of G-lectin protein kinases and receptor-like proteins, each square representing a gene, along with possible downstream signaling (phytohormone) and defense components that are upregulated in *S. lycopersicum* upon attack by *Cuscuta*. SP = *S. pennellii*, SL = *S. lycopersicum*, niS = noninfective stem, SWE = swelling stage, ATT = attaching stage, PEN = penetrating stage, TPM = transcripts per kilobase million.

Our composite datasets further allowed us to compare gene expression in the two *Solanum* hosts to elucidate infection- and defense-specific gene expression profiles. Differential expression analysis within each of the systems revealed that approximately twice as many genes (5,171 vs. 1,956) were upregulated or downregulated in *S. lycopersicum* compared with *S. pennellii* (Fig. 4A). The same clustering approach that was used for *C. campestris* was applied, with a total of 5,889 differentially expressed genes associated with 20 clusters (Supplemental Figs. S5 to S6 and Data Set 4). Several clusters comprised genes that were strongly downregulated exclusively in *S. lycopersicum* either upon first contact (clusters 14, 17) or after attachment and a penetration attempt (clusters 11, 19) (Fig. 4B). Within these clusters, genes related to “cell cycle” and “cytoskeleton organization” and to “photosynthesis” and “gene expression” were strongly overrepresented (Figs. 4, C to E), indicating a reduction in anabolic processes of the host and a switch to homeostasis. In contrast, five clusters comprised genes that were upregulated mainly or only in *S. lycopersicum* either early after contact (clusters 9, 15) or when the parasite was attached and attempted to penetrate (clusters 13, 16, 20). With the exception of cluster 15, whose members showed brief transient increase in transcript abundance when *C. campestris* haustoria were in the attachment stage, none of the other clusters showed pronounced changes in *S. pennellii* (Fig. 4B). Interestingly, the most prominent bins in cluster 9 included protein kinases of the G-lectin family, three of which were located closely in a region on chromosome 2 that was previously linked to resistance to *Cuscuta* (Figs. 4, D and E) (Krause et al. 2018). Cluster 9 further contained the previously identified *CuRe1-like* gene (Solyc08g016210) that may be involved in perceiving *Cuscuta* (Hegenauer et al. 2016; Jhu et al. 2021a), while *CuRe1* (Solyc08g016270) was found in cluster 16 (Figs. 4, F and G). Substantial shifts in expression were also observed in an ACC synthase (*ACS*) gene, two phenylalanine ammonia lyase (PAL) genes, and cytokinin dehydrogenase (CKX) and jasmonic acid oxidase (JOX/JAO) genes (Figs. 4, F and G), consistent with a hypersensitive-like response and increased ethylene biosynthesis and suggesting catabolism of the phytohormones cytokinin and jasmonic acid upon attack.

### *C. campestris* infection triggers changes in cell wall composition in *S. lycopersicum*

**Figure 5.**
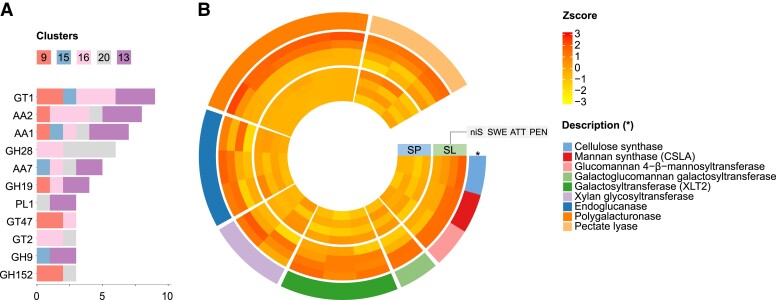
Putative CAZyme genes in *Solanum lycopersicum* cv. M82. **A)** Number of CAZyme motifs per family contained in clustered accessions. Only the most represented CAZyme families are displayed. **B)** Expression pattern of cell wall-related genes assigned to selected CAZyme families, which showed specific upregulation upon *Cuscuta campestris* attack. Values are average z-scores obtained from TPM counts. A z-score value is positive (negative) if the gene expression in a sample type is larger (smaller) than the overall mean expression. Genes are sorted by CAZyme family (outermost layer). SP = *S. pennellii*, SL = *S. lycopersicum*, niS = noninfective stem, SWE = swelling stage, ATT = attaching stage, PEN = penetrating stage, TPM = transcripts per kilobase million.

TBO staining (Fig. 1) suggested a change in cell wall composition at the *S. lycopersicum* infection site, consistent with previous reports (Krause et al. 2018; Jhu and Sinha 2022). Therefore, we focused on carbohydrate-related genes of the two hosts and their expression patterns. Genes from clusters 9, 13, 15, 16, and 20, whose expression levels increased upon exposure to the parasite, were analyzed for the presence of CAZymes. A total of 73 such genes (5.05% of 1446 accessions) from 25 families were identified. Many CAZyme genes that showed altered expression belonged to the GT1 family (for example, flavonol 3-*O*-glycosyltransferases and UDP-glycosyltransferases that might contribute to secondary metabolism with the production of stress-protective anthocyanins and terpene volatiles) (Fig. 5A). Similarly, the expression of many laccases (AA1) and peroxidases (AA2) was found to be induced upon parasitism in *S. lycopersicum*. These enzymes putatively contribute to the deposition of lignin and suberin in attacked shoot tissues, and their upregulation occurred either transiently during early infection (members of clusters 9 and 15) or was sustained throughout the later stages (clusters 13, 16, and 20), which is consistent with the formation of a secondarily modified cell wall involving the phenylpropanoid pathway. Endochitinases (GH19) and thaumatin-like proteins (GH152) can play a role in the accumulation of reactive oxygen species and cell death, and their expression was also detected from the early stage of *Cuscuta* attack (Fig. 5A). In contrast, polygalacturonases (GH28), pectate lyases (PL1), and enzymes involved in mannan synthesis (GT2, GT34) were upregulated only when the parasite had already started to establish itself in the host (Fig. 5, A and B). Galactosyltransferases (GT47) and xylan glycosyltransferases (GT61) may also be involved in the synthesis of xyloglucan and xylan, respectively. Overall, it is noteworthy that genes involved in opposite functions for the same cell wall polymer are induced in the parasite and the host at the same stage of parasitism. This is exemplified by mannan metabolism, where catabolic genes are expressed in *C. campestris* while anabolic functions prevail in *S. lycopersicum*.

### The expression of *C. campestris* mannanases is influenced in a gene- and compound-dependent manner by mannan polysaccharides

**Figure 6.**
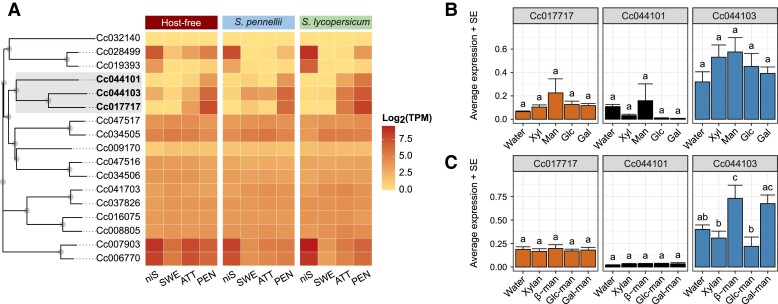
Profiling of the expression of endo-β-1,4-mannanases in *Cuscuta campestris*. **A)** Amino acid sequence-based neighbor-joining tree of putative endo-β-1,4-mannanases shown together with a heatmap of gene expression of the four tissues (niS, SWE, ATT, PEN) in each of the three interaction systems (host-free, on *S. pennellii*, and on *S. lycopersicum*). Accessions selected for the analyses shown in B and C and corresponding branches are highlighted. Colors in the heatmap show average log-transformed TPM in sequenced samples (three biological replicates). **B)** Gene expression of the selected endo-β-1,4-mannanases as measured by RT-quantitative (q)PCR in attaching haustoria. Haustoria was exposed to xylose, mannose, glucose, or galactose in an host-free induction system. The sugars were dissolved in water and applied to filter papers. Values are average gene expression. Error bars indicate SE of the mean (five biological replicates). **C)** Gene expression of the selected endo-β-1,4-mannanases in an host-free induction system supplemented with xylan, β-1,4-mannan, glucomannan, or galactomannan. For each gene (plot panel) in (B) and (C), means with the same letter do not significantly differ (*P* > 0.05 following Tukey multiple pairwise comparisons with BH correction). niS = noninfective stem, SWE = swelling stage, ATT = attaching stage, PEN = penetrating stage, Gal = galactose, Glc = glucose, Xyl = xylose, Man = mannose, β-man = β-1,4-mannan, Glc-man = glucomannan, Gal-man = Galactomannan, TPM = transcripts per kilobase million, SE = standard error.

Overall, our data provided examples of differential induction of genes for cell wall-degrading enzymes in *Cuscuta* during infection and concomitant induction of cell wall modifying or fortifying genes that were more pronounced in the partially resistant *S. lycopersicum* than in *S. pennellii*. This corroborates the importance of cell walls in the *Cuscuta*-host interaction that is highlighted in earlier studies. Remarkably, one set of genes that were induced differentially in *C. campestris* during the three investigated stages was not reported in this context earlier: genes coding for endo-β-1,4-mannanases, three of which were flagged as being induced strongly and early in *Cuscuta* shoots infecting *S. lycopersicum* (Figs. 6A, Supplemental S7). Mannan polysaccharides are wall structural components, and they bind to cellulose along with serving as storage molecules (Voiniciuc 2022). In contrast, mannan oligosaccharides were recently associated with the induction of defense responses (Zang et al. 2019) and were detected in higher amounts in uninfected stems of wild-type *S. lycopersicum* than in several independent susceptible tomato lines introgressed with *S. pennellii* genome fragments (Johnsen et al. 2015). Interestingly, we also found an induction of genes involved in mannan synthesis in *S. lycopersicum* (Fig. 5).

We next investigated whether *C. campestris* detects mannans and, possibly, other cell wall compounds in the host that are present either a priori or appear in an infection-dependent manner and whether it responds accordingly by adjusting the expression of corresponding cell wall-degrading enzymes during an infection. We adapted the existing host-free system and triggered haustorium formation in apical portions of *C. campestris* shoots using far-red light in the presence of different commercial mannan polymers (β-1,4-mannan, glucomannan, and galactomannan) and their sugar monomers (mannose, glucose, and galactose), which may accumulate in infected hosts upon infection. We also included xylan and xylose in our analysis because they have been proposed to play a role in tomato resistance (Johnsen et al. 2015) and associated biosynthetic genes showed differential regulation in *S. lycopersicum* in our transcriptomic data upon attack. Haustorium induced in the presence of the selected cell wall constituents wer examined for the expression of all three *C. campestris* endo-β-1,4-mannanases that were more strongly upregulated during the attaching stage in *S. lycopersicum* (Cc017717, Cc044101, and Cc044103). Although not statistically significant, all three genes showed an increase in expression when exposed to mannose (Fig. 6B, Supplemental Table S3 and Data Set 5); Cc044103 further increased in expression in the presence of all the other tested sugars including xylose (Cc017717: F_(4,20)_ = 1.117, *P* = 0.376; Cc044101: F_(4,20)_ = 1.075, *P* = 0.395; Cc044103: F_(4,20)_ = 1.103, *P* = 0.383). No changes in expression were detected for Cc017717 (F_(4,20)_ = 0.179, *P* = 0.947) and Cc044101 (F_(4,20)_ = 0.666, *P* = 0.623) in the presence of cell wall polymers (Fig. 6C, Supplemental Data Set 6). However, Cc044103 showed statistically significant differences (F_(4,20)_ = 5.608, *P* = 0.003), with an increase in expression following exposure to β-1,4-mannan and galactomannan and decreased expression in the presence of glucomannan, whereas xylan did not have any measurable effects.

**Figure 7.**
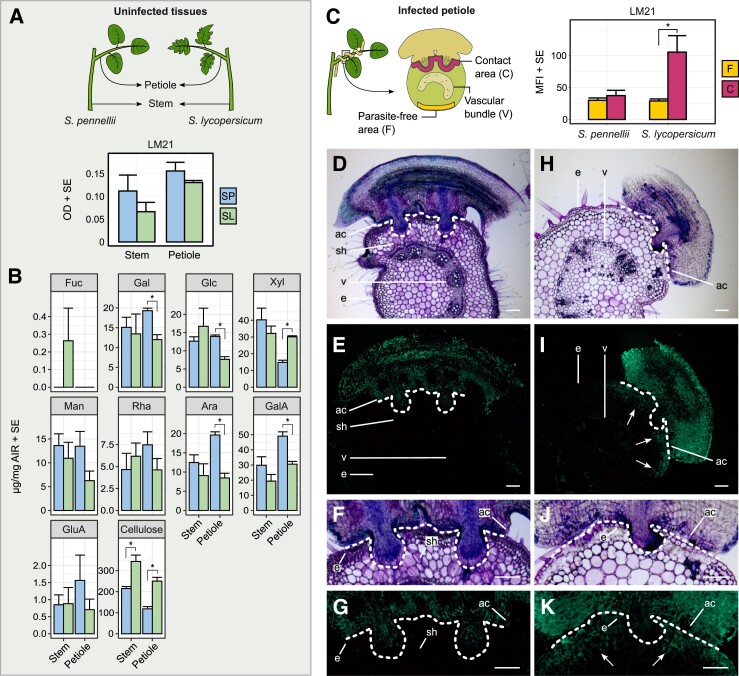
Cell wall composition of tomato hosts. **A)** Mannan epitope detection using the LM21 antibody in AIR extracts from uninfected stems and petioles of *Solanum pennellii* versus *S. lycopersicum*. Values are average OD_450_ from the ELISA assay. Error bars indicate SE of the mean (two technical replicates, each sample being a pool of five stems or petioles from different plants). **B)** Quantification of matrix sugars in uninfected stems and petioles of *S. pennellii* versus *S. lycopersicum*. Values are average sugar amounts (µg) per mg of dry AIR. Error bars indicate SE of the mean (three biological replicates). For each sugar and tissue type, means with an asterisk significantly differ between the two tomato species following a Student's *t*-test (*P* ≤ 0.05). **C)** MFI in infected petioles at the contact region with the parasite versus a parasite-free area as measured from Toluidine Blue O (TBO)-stained cross sections using the LM21 antibody for (galacto)(gluco)mannan epitope detection. Error bars indicate SE of the mean (five biological replicates). Means with an asterisk significantly differ following a Student's *t*-test (*P* ≤ 0.05). **D, E**, **F, G)** Distribution of mannan epitopes in a *C. campestris* infection site on an *S. pennellii* petiole using the LM21 antibody. The pictures show a TBO-stained cross section and corresponding immunofluorescence. Dotted lines delineate the interface at the contact region between parasite and host. **H, I**, **J, K)** Distribution of mannan epitopes in a *C. campestris* infection site on an *S. lycopersicum* petiole. The detection of mannan epitopes in the host petiole underneath the attachment ring (arrows) indicates an accumulation of mannan polymers in response to attempted parasitism. Scale bars are 200 *µ*m. SP = *S. pennellii*, SL = *S. lycopersicum*, Fuc = fucose, Gal = galactose, Glc = glucose, Xyl = xylose, Man = mannose, Rha = rhamnose, Ara = arabinose, GalA = galacturonic acid, GluA = glucuronic acid, ac = adhesive cell, e = petiole epidermis, sh = searching hyphae, v = petiole vascular bundle, AIR = alcohol insoluble residue, SE = standard error, MFI = mean fluorescence intensity.

In order to investigate constitutive differences among cell wall components—particularly mannans—between stems or petioles of uninfected *S. lycopersicum* and *S. pennellii* plants, we first performed an enzyme-linked immunosorbent assay (ELISA) using the LM21 monoclonal antibody, which recognizes mannan epitopes (Johnsen et al. 2015). This revealed no clear interspecies differences in (hetero)mannans for each tissue type (Fig. 7A). This was supported by a wider analysis of cell wall extracts from stems and petioles using differential chemical digestion and monosaccharide profiling, which indicated insignificant variation between the samples of mannose (stem: t_(4)_ = 0.642, *P* = 0.556; petiole: t_(4)_ = 1.950, *P* = 0.123), rhamnose (stem: t_(4)_ = 0.633, *P* = 0.561; petiole: t_(4)_ = 1.449, *P* = 0.221), and glucuronic acid (stem: t_(4)_ = 0.057, *P* = 0.957; petiole: t_(4)_ = 1.067, *P* = 0.346) in contrast to cellulose (stem: t_(4)_ = 4.010, *P* = 0.016; petiole: t_(4)_ = 6.435, *P* = 0.003) and xylose (stem: t_(4)_ = 0.981, *P* = 0.382; petiole: t_(4)_ = 10.460, *P* < 0.001), which were elevated significantly in one or both *S. lycopersicum* tissues, and galactose (stem: t_(4)_ = 0.293, *P* = 0.784; petiole: t_(4)_ = 5.096, *P* = 0.007), glucose (stem: t_(4)_ = 0.795, *P* = 0.471; petiole: t_(4)_ = 7.173, *P* = 0.002), arabinose (stem: t_(4)_ = 0.932, *P* = 0.404; petiole: t_(4)_ = 7.173, *P* = 0.002), and galacturonic acid (stem: t_(4)_ = 1.464, *P* = 0.217; petiole: t_(4)_ = 5.321, *P* = 0.006), which were present in significantly lower amounts (Fig. 7B). A comparison of the infection sites on both hosts by immunolabelling showed that mannan levels were higher in the petiole sections closest to the infection sites in *S. lycopersicum*, but not in *S. pennellii* (Figs. 7, C to K, Supplemental Fig. S8), which is consistent with the expression patterns of mannan biosynthesis genes in both hosts. The fluorescence intensity under the attachment ring in *S. lycopersicum* compared to parasite-free areas was significantly higher (t_(8)_ = 4.902, *P* = 0.001), which was not the case in *S. pennellii* (t_(8)_ = 0.711, *P* = 0.497) (Fig. 7C).

## Discussion

It is widely accepted that haustorium formation can be triggered by signals that are not host-dependent, including light and tactile stimuli (Tada et al. 1996; Haidar 2003; Olsen et al. 2016b; Kaga et al. 2020). Additionally, the relatively broad host range of many *Cuscuta* species, including *C. campestris*, and the scarcity of resistant plants suggest that the parasite may rely on a conserved infection strategy that is targeted to exploit weaknesses common to all hosts. However, the results of our study, which were based on a comparison of transcriptional profiling of infection sites from two relatively closely related hosts from the *Solanum* genus, indicate that *C. campestris* can sense specific molecules in a given host and adjust their infection strategy accordingly, which involves changes in gene expression. Of the three stages of haustoriogenesis, the swelling stage showed the most extensive overlap in patterns of gene expression between host-free and host-exposed systems, indicating that the initial inducing signals are more physical than biological in nature. The genes that were differentially expressed in this stage are mainly associated with cell metabolism and proliferation. Some showed sustained levels of expression in later stages of haustorium development, and these included genes encoding fasciclin-like arabinogalactan (FLA) proteins, whose expression is developmentally regulated in both the haustorium holdfast and hyphal cells, which are both in intimate host contact (Hozumi et al. 2017). In contrast, many of the genes whose expression was increased in both host-exposed systems were activated in the subsequent attaching and penetrating stages. Some of them, like AS2/LOB transcription factors, are known key regulators of plant organ development (Xu et al. 2016). In *Cuscuta*, CcLBD25 is suggested to play a role in cell wall loosening and in forming vascular connections (Jhu et al. 2021b), which reflects the physical changes that the haustorium undergoes during this stage of infection. This is consistent with recent literature and with the idea that haustorium development in parasitic plants is only fully achieved upon the perception of host-derived signals. In this regard, Kaga et al. (2020) showed that genes involved in the development and proliferation of vascular stem cells in *C. campestris* were expressed during haustoriogenesis even in the absence of a host, whereas the connection between search hyphae and the host xylem was required to activate some key genes in the parasite to enable differentiation into nonautonomous vessel cells. Narukawa et al. (2021) further demonstrated that *C. campestris* perceives host-produced ethylene, which promotes the elongation of search hyphae. Along this line, we found a differential upregulation of *ACO* genes in *C. campestris* parallelly with a pronounced accumulation of *ACS* gene transcripts in *S. lycopersicum* in the two later infection stages, which may indicate that ACC is transported from the host to the parasite and then converted to ethylene, which may act on the parasite either in addition to or instead of host-derived ethylene. Concomitantly, reduced transcriptional activity in *S. lycopersicum* genes coding for cyclins, kinases, and other regulatory proteins, which are essential for the onset and progression of mitosis, was triggered upon contact with swelling haustoria, suggesting growth inhibition. Cell cycle modulation is known to play a role in the adaptation of plants to abiotic and biotic stresses (Qi and Zhang 2019). Such repression of genes for cyclins, kinases, and other proteins involved in mitosis was not evident in *S. pennellii*, suggesting that either the parasite is able to suppress such reactions or that it is not perceived as an intruder for other reasons. An obvious reason could be similarities in cell wall composition because it is the foremost barrier that invading species must breach.

In contrast to the considerable knowledge on the degradation of host cell walls by fungal plant pathogens (Kubicek et al. 2014; van der Does and Rep 2017), the modulation of cell wall-degrading genes at the transcriptional level by *Cuscuta* is still poorly documented. Cellulose is an important structural component of the primary cell wall and forms a complex matrix with pectins and hemicelluloses, such as xyloglucan, xylan, and mannan (Gigli-Bisceglia et al. 2020). In our study, transcripts of several genes coding for cell wall-degrading enzymes, including endoglucanases, pectate lyases, xylanases, and mannanases, were found to be highly abundant in the final infection stages of *Cuscuta,* which is consistent with previous reports on the detection of such enzymes in haustoria (Nagar et al. 1984; Johnsen et al. 2015). That many of these genes were upregulated more strongly when *C. campestris* infected *S. lycopersicum* than *S. pennellii* is most likely causally connected with the concomitant induction of cell wall synthesis-associated genes in *S. lycopersicum*. Infection with the species *C. reflexa* was previously observed to trigger cell wall remodeling in infection sites of *S. lycopersicum* with an accumulation of xyloglucans, pectins, and arabinogalactan proteins (Krause et al. 2018). Moreover, susceptible interspecific *S. lycopersicum × S. pennellii* introgression lines (ILs) showed some characteristic reductions in hemicellulosic compounds in uninfected stem parts of previously infected plants, suggesting that constitutive differences in cell wall composition contribute to the different outcomes of an infection (Johnsen et al. 2015). However, while our analysis of mono- and polysaccharides in cell wall extracts from never-infected hosts showed some constitutive differences between *S. lycopersicum* and *S. pennellii*, particularly with respect to cellulose, overall, these differences were not very pronounced, suggesting that a parasite infection is necessary to trigger wall remodeling. Accordingly, a differential accumulation of mannans was shown to occur postinfection in *S. lycopersicum* but not in *S. pennellii* (Fig. 7). These cell wall fortifications in *S. lycopersicum* might be perceived by *Cuscuta* and trigger the observed expression of genes for cell wall-degrading enzymes, including three mannanase genes. Such a hypothetical modulation of the parasite's enzymatic profile in response to the cellular environment they encounter was corroborated by in vitro studies of the transcriptional responsiveness of *Cuscuta* to selected purified mono- and polysaccharides, where mannose in its monomeric form or as part of linear or branched chains triggered the expression of at least one mannanase gene. Knowledge of the role of mannans in plants, while recently expanded, is still limited. However, it is thought that branched galactomannan, one of the two polysaccharides that triggered expression of Cc044103 strongly when applied alone, predominantly has storage functions, while linear homomannan, being the second type with a similar effect on the *Cuscuta* gene, plays cell wall structural roles (Singh et al. 2018). Moreover, homomannans were reported to give rise to mannan oligosaccharides, which were observed in rice (*Oryza sativa*) and *Nicotiana benthamiana* to act as DAMPs and activate defense responses (Zang et al. 2019). It is not possible to distinguish between the different mannans with the available antibodies, but based on the functions of known mannan polymer types, we hypothesize that the mannan-related epitope that accumulated in *S. lycopersicum* as a result of *Cuscuta* infection reflects linear homomannan that serves the host in fortifying its cell walls or in signaling a breach in cell wall integrity.

Intriguingly, our data provide evidence for the role of mannans in the interaction: a differential expression of surface-localized putative lectin receptor-like kinases (lecRLKs) of the G-family in tomato (Fig. 4). G-type lecRLKs contain a domain with reported binding affinity to mannose and are reported to participate in stress responses and resistance to pathogens (Sun et al. 2020). Thus, the upregulation of mannans in the damaged tissue below the infection site in *S. lycopersicum* may represent an increase in the potential source of DAMPs. This idea is supported by the fact that three of the genes encoding the lecRLK receptors were localized in the same region on chromosome 2 that was previously linked to resistance to *Cuscuta* using tomato ILs (Krause et al. 2018). The observation that the same ILs had lower levels of mannans than *S. lycopersicum* M82 (Johnsen et al. 2015) is consistent with the cell walls of hosts providing a signal for the *Cuscuta* parasite to optimize its infection approach. This would also explain the upregulation of genes coding for berberine bridge enzyme-like proteins in the parasite, which are shown to oxidize (and, thus, inactivate) oligogalacturonides (OGs) and cellodextrins (CDs) (Benedetti et al. 2018; Locci et al. 2019). This occurs toward the later stages of haustorium development and suggests that *Cuscuta* tries to actively modulate the activity or abundance of DAMPs to infect the host.

The various observed transcription profiles in the parasite and its two hosts indicate that host defense and the parasitic attack involve multiple layers of control. As the haustorium grows, different cellular metabolites and polysaccharide fragments generated as a consequence of cell wall remodeling or deconstruction accumulate at the host/parasite interface, depending on the physiological state of the host and possibly that of the parasite. The synthesis of homogalacturonan and mannan polymers, together with FLA proteins during *C. campestris* parasitism as indicated by expression data, and the detection of mannans in adhesive cells of the attachment ring by immunolabelling, are consistent with such processes. Determining the exact mechanisms responsible for the immune response in cultivated tomato, their interplay, and the extent to which the parasite interferes with them throughout parasitization, may help with engineering crops that have multilayered resistance. However, the ability of the parasite to fine-tune its infection strategy to the encountered host needs to be understood, including whether this applies only to those species with a broad host range. We note that while the cell wall antibodies used here for immunolocalization in infection sites do not distinguish different mannan types and the host-free system does not take the natural biological environment encountered by the parasite into account, they are readily applicable to other *Cuscuta* species and, collectively, the results provide insights to how hosts and parasites influence each other. More sophisticated host-free experimental systems, such as that recently described by Bernal-Galeano et al. (2022), may help to further elucidate causal connections between wall composition and regulation of the different CAZyme genes in *Cuscuta* and their importance for the success of *Cuscuta* parasites.

## Materials and methods

### Plant materials

Plants were maintained at the Climate Laboratory of the Arctic University of Norway, Tromsø, in a greenhouse under 24 h light and approximately 21 °C. Field dodder (*Cuscuta campestris*) was obtained from the Botanical Garden of the University of Kiel (Germany) and propagated on horseshoe geranium (*Pelargonium zonale*). Cultivated tomato (*Solanum lycopersicum*) cv. M82 and *S. pennellii* (LA0716) were obtained from the C.M. Rick Tomato Genetics Resource Center (TGRC) as seeds and were grown in sphagnum peat (Veksttorv, Tjerbo, Norway) mixed at a 2:1 (v/v) ratio with perlite (Agra-perlite, PULL Rhenen, The Netherlands).

### Sampling of infection sites

Distal portions of *C. campestris* shoots, including tips (∼15 cm), were harvested from *P. zonale* and attached to the leaf petioles of either *S. lycopersicum* or *S. pennellii* individuals (5- to 6- week-old). A continuous 16 h light, 2 h far-red light, and 6 h dark regime was applied to promote twining and infection by the parasite. Infection sites, consisting of *Cuscuta* coils and the entwined host tissues, were excised using razor blades and frozen individually in liquid nitrogen. Noninfective *C. campestris* stem sections were further mixed with uninfected *S. lycopersicum* or *S. pennellii* petiole sections to create chimeric reference samples and frozen. Individual samples containing both parasite and host tissues were assigned to one of the stages of haustorium development (noninfective, swelling, attaching, or penetrating) before probing for marker gene expression by reverse transcription (RT)-quantitative (q)PCR following Bawin et al. (2022) using Cc028378/Cc002986 as references. Triplicates of each stage (including noninfective samples) and host-parasite pairs were selected for mRNA sequencing, rendering a total of 24 samples.

### RNA extraction, library preparation, and sequencing

The same pipelines described earlier (Bawin et al. 2022) were used except that the Maxwell 16 low elution volume Plant RNA Kit and Maxwell 16 Instrument (Promega) were used for RNA extraction instead. Approximately 100 mill paired-end reads (150 bp in length) per sample were produced on a Illumina HiSeq4000 sequencing platform.

### Read mapping and quantification

Paired-end reads (101 bp) from the host-free experiment in Bawin et al. (2022) (NCBI, PRJNA666991) were processed along with the composite host-parasite tissue samples. Quality assessment was performed using FASTQC v. 0.11.8 (https://www.bioinformatics.babraham.ac.uk) with default parameters. Trimming was performed using Trimmomatic v. 0.32.1 (Bolger et al. 2014) with ILLUMINACLIP:TruSeq3-PE.fa:2:30:10, SLIDINGWINDOW:4:20, and MINLEN:135 (85 for reads from the host-free experiment). Mapping was performed using STAR v. 2.7.5 (Dobin et al. 2013) with default parameters, except a –sjdbOverhang of 149 (100 for reads from the host-free experiment), on a chimeric assembly made of *C. campestris* r0.32 (Vogel et al. 2018) and *S. lycopersicum* SL4.0 (https://solgenomics.net) genomes that were concatenated. Uniquely mapped read pairs (fragments) were counted using featureCounts v. 1.6.4. (Liao et al. 2014) on the exons of the total gene models. All analyses were run on the open web-based platform Galaxy Europe (Afgan et al. 2022; https://usegalaxy.eu). Parasite and host libraries were then split, and counts were normalized into Transcripts Per Kilobase Million (TPM).

### Differential expression analysis

Differentially expressed genes were identified between sample groups using DESeq2 v. 1.34.0 (Love et al. 2014) with raw read counts as input data. *P*-values were adjusted using the Benjamini–Hochberg (BH) procedure (Benjamini and Hochberg 1995). A false discovery rate (FDR) cutoff of 0.05 and a minimum absolute log_2_ fold change (|log_2_(FC)|) of 1.5 were used to retain genes of interest.

### Soft clustering

The fuzzy c-means algorithm, as implemented in Mfuzz v. 2.54.0 (Futschik and Carlisle 2005), was used to cluster based on their log_2_(TPM) values genes that: (i) were differentially expressed in at least one of any possible pairwise comparison of stages, and (ii) had a minimum of five normalized counts in at least three samples of the same interaction system. For each cluster, the trait (pattern of expression) with the best fit was determined by a point-biserial correlation test between cluster center value and all possible combinations of stages across host-free and host-exposed systems (with 1 indicating expression in one or several stages and 0 indicating no expression).

### Gene annotation and enrichment analysis

Assignment of *C. campestris* genes to MapMan4 v.4.0 functional categories was performed using the online Mercator annotation tool (Schwacke et al. 2019). Gene set enrichment analyses were performed by applying a hypergeometric test, with redundant soft clusters (that is, assigned to the same trait) merged. *P*-values were adjusted using the BH procedure. Bins with an FDR ≤ 0.05 were considered significantly enriched.

### Identification of CAZyme motifs

Protein sequences from the *C. campestris* r0.32 and *S. lycopersicum* SL4.0 genomes were searched against the dbCAN Hidden Markov Model (HMM) database (v. 10) through the dbCAN2 meta server (Zhang et al. 2018) to identify CAZymes and associated carbohydrate-binding modules. For each *C. campestris* gene, the variant with the longest sequence was selected as representative. Only HMM profiles with a coverage >0.35 and an e-value <1e-15 (default thresholds on server) were retained.

### Enzyme-linked immunosorbent assay

Alcohol insoluble residues (AIR) were extracted from a pool of stems or petioles of five different uninfected *S. pennellii* and *S. lycopersicum* plants. Cell wall components were sequentially extracted from lysed lyophilized samples by sequentially adding 1 mL solutions of 70% v/v, 80% v/v, 90% v/v, and 100% v/v EtOH, followed by 100% v/v acetone and methanol:chloroform (2:3). Residues were further extracted using 4 M KOH containing 1% w/v NaBH_4_. AIR extracts were diluted 1:10 in phosphate-buffered saline (PBS), neutralized to a pH between 6.5 and 7.5 with 80% v/v acetic acid, and coated directly onto microtiter ELISA plates (NUNC Maxisorp, Thermo Fisher Scientific, Denmark) in duplicates. Mannan was detected using the monoclonal antibody LM21 (PlantProbes) following the method described in Cornuault et al. (2014). Plates were read on a microplate reader (Agilent Biotek, Gen5 software) for optical density (OD) values at 450 nm.

### Quantification of cellulose and matrix sugars

Stems (two areas from a side branch) and petioles (the uppermost part) from the two tomato species were collected in triplicates and flash frozen in liquid nitrogen before lyophilization. The dried material was snap-frozen again and homogenized using steel balls in a tissuelyzer (MM 400 ball mill (Retsch)). AIR extracts, Saeman, and matrix hydrolysis samples were prepared as in Yeats et al. (2016) before analysis using high-performance anion-exchange chromatography with pulsed amperometric detection (HPAEC-PAD) on a Dionex ICS3000 (Thermo Fisher) equipped with a CarboPac PA-20 column (Thermo Fisher). Elution was performed with 3 mM NaOH in order to resolve and quantify Fuc, Gal, Glc, Xyl, and Man, and with 18 mM NaOH to resolve and quantify Rha and Ara. A 100 mM NaOH and a 10-min 50 to 200 mM sodium acetate gradient were used to resolve and quantify GalA and GlcA. The quantities of matrix sugars and cellulose were determined as in Yeats et al. (2016) and compared with a Student's *t*-test (*P* ≤ 0.05).

### Immunolabelling and differential staining of infection sites

Production of 70 *µ*m vibratome sections in infection sites and TBO staining of these were carried out as described in Bawin et al. (2022). Immunolabeling of fresh sections was done before TBO staining as described in Olsen et al. (2016b) with some modifications: incubation with the primary antibody LM21 (PlantProbes) (diluted 1:10 in blocking buffer) was carried out overnight at 4 °C and Alexa Fluor 488 Goat antirat IgG (Invitrogen) (diluted 1:200 in blocking buffer) was used as a secondary antibody. Micrographs were taken using an Axio Zoom.V16 with a Plan-NEOFLUAR Z 2.3 × objective and an Axiocam 305 color camera (all from Zeiss). Fluorescence images were taken with a green fluorescent protein filter set (450 to 490 nm excitation wavelength, 500 to 550 nm emission wavelength) with 200 to 400 ms exposure times. Mean Fluorescence Intensity (MFI) in the micrographs from immunolabelled sections was inferred from measurements with Fiji v. 2.3.0 (Schindelin et al. 2012) using the Measure function from the Analyze menu after “rolling ball” background subtraction. Statistical significance was based on images from five independent replicates (infection sites) per host species. For each host species, fluorescence intensity in those areas in contact with the parasite and parasite-free areas were compared with a Student's t-test (*P* ≤ 0.05). Profiling of fluorescence intensity was achieved using the Plot profile function from the Analyze menu after “rolling ball” background subtraction.

### Haustorium induction in the presence of commercial compounds

Induction of *C. campestris* haustoria was carried out as described (Bawin et al. 2022) but with a glass-fiber filter paper (Whatman GF/F, 90 mm) between *Cuscuta* and the Petri dish that was soaked beforehand with 4 ml of a 2 mg/ml suspension of either xylose (Tokyo Chemical Industry, CAS 58-86-6), mannose (Thermo Fischer Scientific, CAS 3458 to 3428-4), glucose (Sigma Aldrich, CAS 50-99-7), galactose (Tokyo Chemical Industry, CAS 59-23-4), xylan (Megazyme, CAS 9014-63-5), β-1,4-mannan (Megazyme, CAS 9036-88-8), glucomannan (Megazyme, CAS 11078-31-2), or galactomannan (Megazyme, CAS 11078-30-1) in water and subsequently dried. Ten visually similar sites from three or more individual stems were excised and pooled before being frozen in liquid nitrogen. Five biological replicates were harvested for each modality and tested by RT-qPCR for the expression of three endo-β-1,4-mannanase genes (Cc017717, Cc044101, Cc044103) following Bawin et al. (2022) with Cc028808/Cc006757 as references. For each gene, expression values were compared between treatments using a generalized linear model and Tukey multiple pairwise comparisons with BH correction (*P* ≤ 0.05).

### Accession numbers

Sequence data from this article can be found in the GenBank/EMBL data libraries under accession numbers VFR03631 (Cc017717), VFQ62408 (Cc044101), and VFQ62410 (Cc044103).

## Supplementary Material

kiad505_Supplementary_Data

## Data Availability

The generated raw reads from this article can be found in the NCBI Sequence Read Archive (SRA) under the accession number PRJNA860997. Expression data are available upon reasonable request.

## Supplemental data

The following materials are available in the online version of this article.

**Supplemental Data Set 1.** RT-qPCR expression of markers of haustorium development in *C. campestris* versus *S. lycopersicum* cv. M82.

**Supplemental Data Set 2.** RT-qPCR expression of markers of haustorium development in *C. campestris* versus *S. pennellii* cv. LA0716.

**Supplemental Data Set 3.** Soft clustering of gene expression in *C. campestris*.

**Supplemental Data Set 4.** Soft clustering of gene expression in tomato hosts.

**Supplemental Data Set 5.** RT-qPCR expression of selected endo-β-1,4-mannanases in *C. campestris* exposed to monosaccharides.

**Supplemental Data Set 6.** RT-qPCR expression of selected endo-β-1,4-mannanases in *C. campestris* exposed to polysaccharides.

**Supplemental Figure S1.** Expression profile of stage-specific marker genes of haustorium development in samples selected for sequencing.

**Supplemental Figure S2.** Hierarchical clustering of sequenced biological replicates.

**Supplemental Figure S3.** Soft clustering of gene expression in *C. campestris*.

**Supplemental Figure S4.** Gene expression profiles (*C. campestris*) in soft clusters.

**Supplemental Figure S5.** Soft clustering of gene expression in tomato hosts.

**Supplemental Figure S6.** Gene expression profiles (tomato hosts) in soft clusters.

**Supplemental Figure S7.** Expression profile of selected endo-β-1,4-mannanases in *C. campestris*.

**Supplemental Figure S8.** Profiling of fluorescence intensity in tomato infection sites.

**Supplemental Table S1.** Quality filtering and trimming statistics.

**Supplemental Table S2.** Mapping statistics.

**Supplemental Table S3.** Primer sequence pairs for selected endo-β-1,4-mannanases with their amplicon sizes.
