# Supplementary material for: Cuscuta campestris fine-tunes gene expression during haustoriogenesis as an adaptation to different hosts
Source: Plant Physiol. 2023 Sep 14;194(1):258–73. doi: 10.1093/plphys/kiad505 (PMC10756757; doi:10.1093/plphys/kiad505)
Supplement: kiad505_Supplementary_Data [file kiad505_supplementary_data.zip › PP2023RA01224D_Supplemental_Material.pdf]

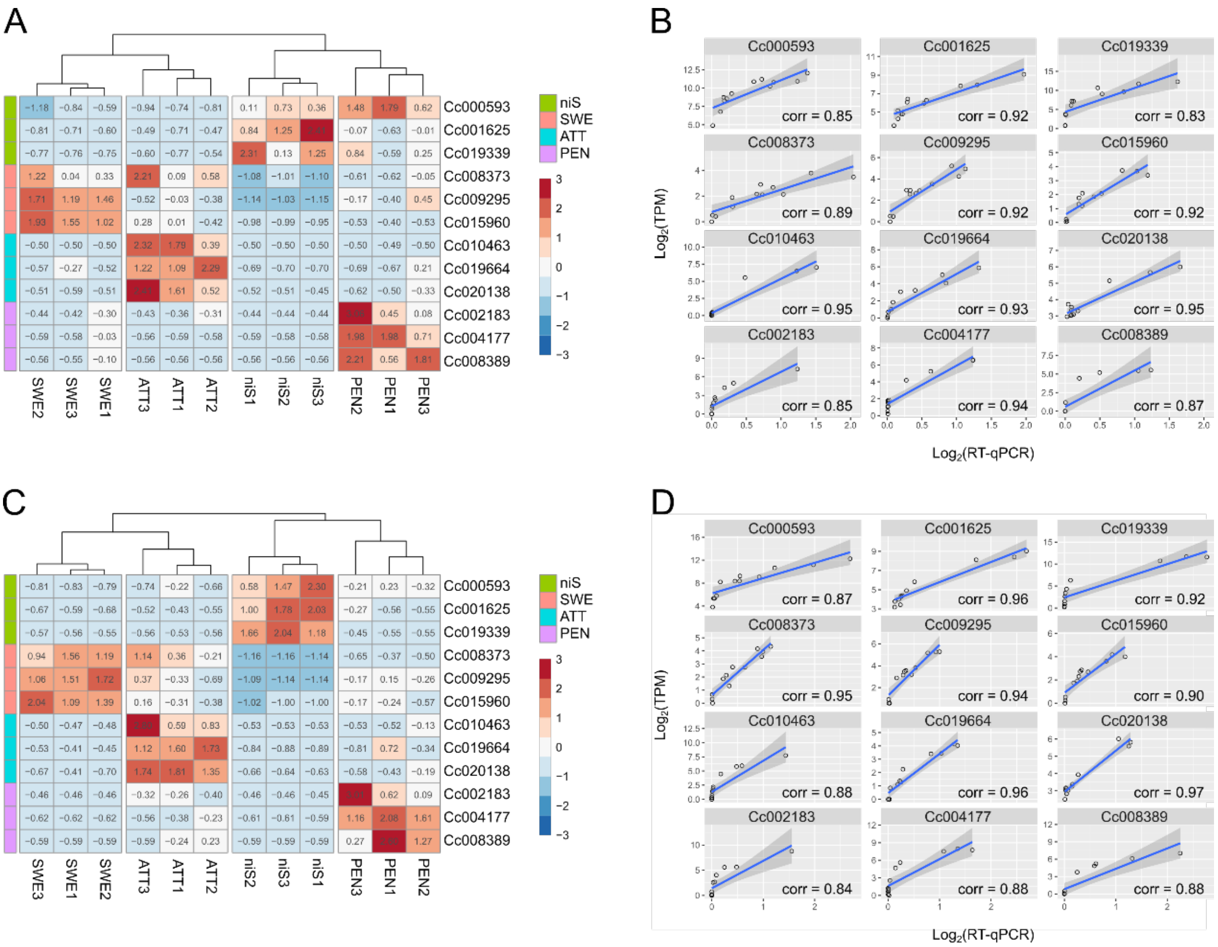

Supplemental Figure S1. Expression profile of stage-specific marker genes of haustorium development in samples selected for sequencing. (A) Reverse transcription (RT)-quantitative (q)PCR gene expression measured in *Cuscuta campestris* growing on *Solanum pennellii* (LA0716). Expression values were normalized against Cc002986 and Cc028378 as in Bawin et al. (2022), and transformed into z-scores. Hierarchical clustering of the samples was based on Euclidean distance. A z-score value is positive (negative) if gene expression in a sample is larger (smaller) than the mean row expression. (B) Validation of the sequencing data. Pearson correlation coefficients (corr) between logarithm-transform "Transcripts Per Kilobase Million" (TPM) values and logarithm-transform normalized RT-qPCR gene expression in sequenced samples (triplicates) are provided in each plot panel with 95% confidence interval (shaded areas). (C) RT-qPCR gene expression measured in *C. campestris* growing on *S. lycopersicum* cv. M82. (D) Validation of the sequencing data. niS = non-infective stem, SWE = swelling stage, ATT = attaching stage, PEN = penetrating stage.

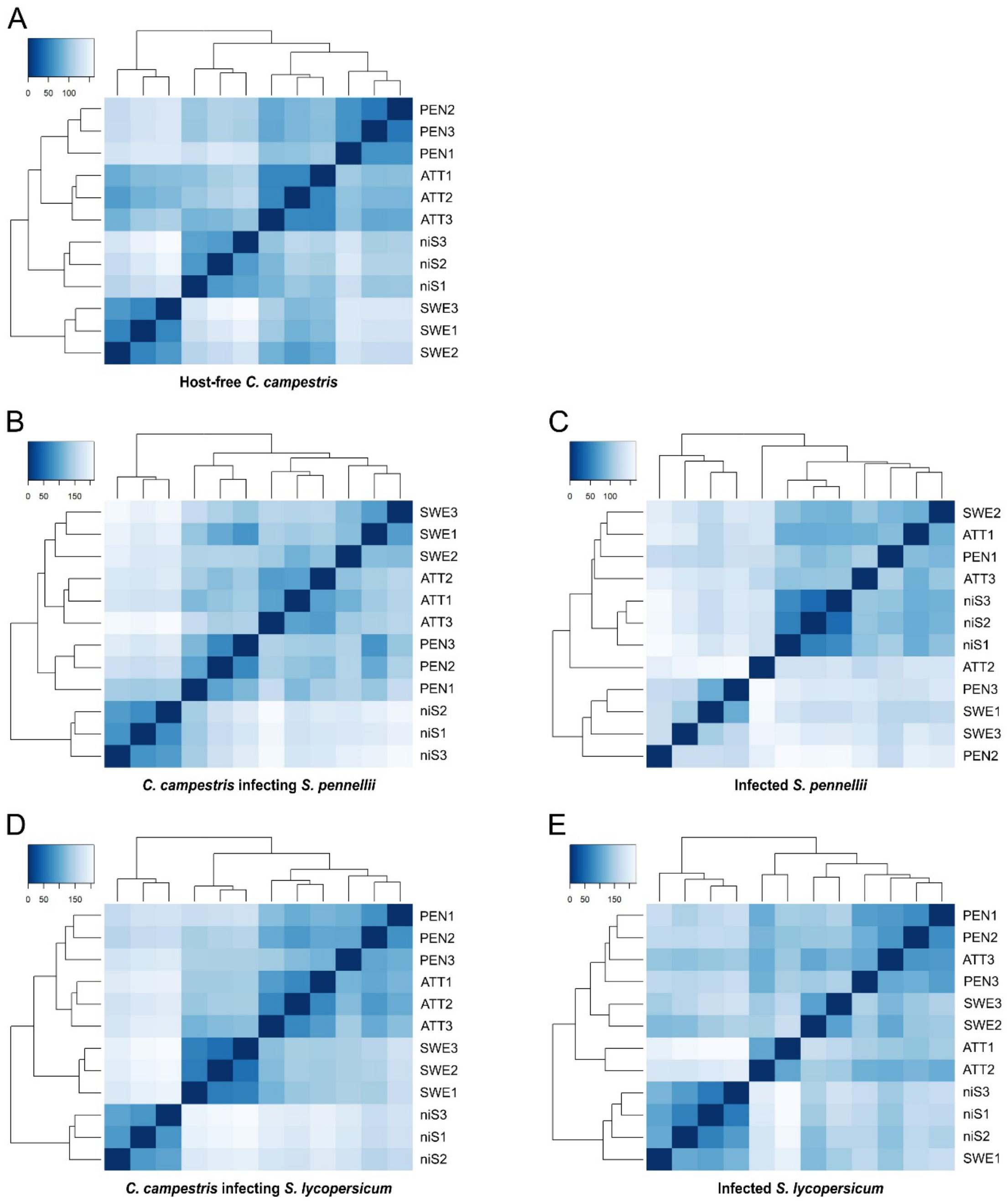

Supplemental Figure S2. Hierarchical clustering of sequenced biological replicates. Mapping of transcriptome sequencing reads from individual infection sites was performed on a chimeric assembly of the *Cuscuta campestris* and *Solanum lycopersicum* genomes. Parasite and host libraries were analyzed separately. Reads from the host-free experiment in Bawin *et al.* (2022) (NCBI, PRJNA666991) were processed the same way. (A) *C. campestris* in a host-free experimental setup. (B) *C. campestris* infecting *S. pennellii*. (C) Infected *S. pennellii*. (D) *C. campestris* infecting *S. lycopersicum*. (E) Infected *S. lycopersicum*. Calculations are based on Euclidean distance between $\log_2$ (counts-per-million + pseudocount) values. The map is symmetrical because the samples are plotted against each other. The darker the color, the closer the samples. niS = non-infective stem, SWE = swelling stage, ATT = attaching stage, PEN = penetrating stage.

A

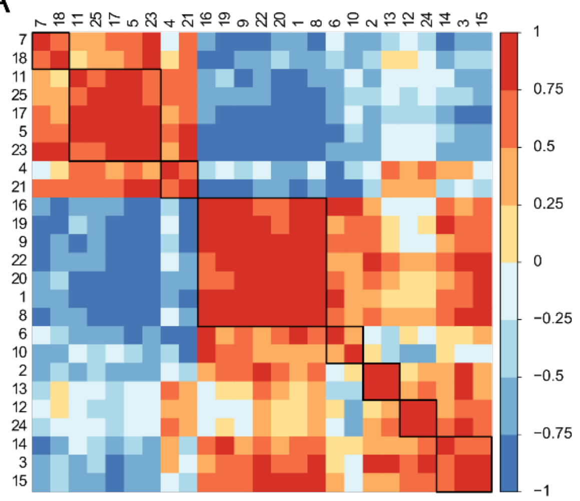

B

	Host-free				*S. pennellii*				*S. lycopersicum*				
	niS	SWE	ATT	PEN	niS	SWE	ATT	PEN	niS	SWE	ATT	PEN	
Cluster 7	0.48	-0.73	-0.41	-0.41	1.69	0.04	-0.37	0.38	1.28	-0.85	-0.72	-0.40	corr = 0.87, p < 0.001
Cluster 18	-0.27	-0.88	-0.39	-0.58	1.26	-0.85	-0.71	0.00	1.78	-0.47	0.32	0.80	corr = 0.89, p < 0.001
Cluster 11	1.35	0.10	0.93	0.89	0.04	-1.06	-0.58	-0.06	0.66	-1.54	-0.62	-0.11	corr = 0.82, p = 0.001
Cluster 25	0.28	-0.73	0.63	1.40	1.00	-1.35	0.16	-0.19	0.81	-0.64	-0.76	-0.61	corr = 0.88, p < 0.001
Cluster 17	1.26	-0.05	0.59	0.43	1.06	-0.86	-0.73	-0.29	1.23	-1.20	-0.95	-0.50	corr = 0.91, p < 0.001
Cluster 5	1.01	-0.37	0.57	0.58	0.79	-1.50	-0.65	-0.16	1.31	-1.52	-0.35	0.27	corr = 0.86, p < 0.001
Cluster 23	0.79	-0.66	0.05	0.17	1.56	-1.12	-0.68	-0.06	1.51	-1.24	-0.42	0.09	corr = 0.85, p < 0.001
Cluster 4	0.05	-0.70	0.56	1.51	-0.64	-1.06	-0.01	0.13	0.17	-1.02	0.23	0.79	corr = 0.83, p = 0.001
Cluster 21	0.36	-1.16	-0.13	0.68	0.75	-0.93	-0.26	0.36	0.85	-1.60	0.26	0.81	corr = 0.89, p < 0.001
Cluster 16	-0.64	1.27	0.18	-0.33	-1.17	1.38	0.16	-0.20	-1.55	1.30	-0.08	-0.31	corr = 0.84, p = 0.001
Cluster 19	-0.61	0.95	0.53	0.00	-1.78	0.61	0.53	-0.17	-1.52	0.98	0.45	0.03	corr = 0.87, p < 0.001
Cluster 9	-1.05	0.93	0.06	-0.72	-1.15	0.71	0.85	-0.46	-1.23	1.40	0.87	-0.21	corr = 0.90, p < 0.001
Cluster 22	-1.24	0.36	-0.12	-0.31	-1.42	0.36	0.90	-0.02	-1.37	1.04	1.24	0.59	corr = 0.87, p < 0.001
Cluster 20	-1.38	0.25	-0.63	-0.90	-0.62	0.96	0.60	0.24	-1.09	1.45	0.82	0.29	corr = 0.90, p < 0.001
Cluster 1	-1.18	0.76	-0.34	-0.60	-1.00	1.39	0.14	0.23	-1.41	1.49	0.25	0.28	corr = 0.85, p < 0.001
Cluster 8	-1.16	0.54	-0.18	-0.28	-1.56	1.01	0.54	0.41	-1.50	1.19	0.59	0.42	corr = 0.90, p < 0.001
Cluster 6	-0.74	0.84	-0.50	-0.76	0.12	1.63	-0.57	0.26	-0.81	1.19	-0.53	-0.13	corr = 0.89, p < 0.001
Cluster 10	0.13	1.34	0.79	-0.29	-0.62	0.48	-0.01	-0.83	-0.63	0.98	-0.43	-0.92	corr = 0.88, p < 0.001
Cluster 2	-0.83	0.02	0.00	-0.43	-0.81	-0.32	1.47	-0.57	-0.92	0.46	1.82	0.11	corr = 0.87, p < 0.001
Cluster 13	-0.66	-0.80	-0.16	0.36	-0.60	-0.72	1.12	-0.08	-0.49	-0.63	1.78	0.88	corr = 0.90, p < 0.001
Cluster 12	-0.61	-0.91	-0.68	0.10	-0.29	0.34	-0.36	1.30	-0.44	-0.67	0.45	1.77	corr = 0.86, p < 0.001
Cluster 24	-0.80	-0.88	-0.32	1.23	-0.91	-0.10	-0.07	0.96	-0.76	-0.67	0.78	1.53	corr = 0.93, p < 0.001
Cluster 14	-0.24	0.24	0.23	0.80	-1.96	0.22	0.04	0.42	-1.25	0.22	0.39	0.89	corr = 0.91, p < 0.001
Cluster 3	-1.18	-0.41	-0.33	0.30	-1.24	0.02	0.91	0.48	-1.21	0.22	1.37	1.08	corr = 0.86, p < 0.001
Cluster 15	-1.09	-0.29	-0.55	0.07	-1.10	0.99	0.02	1.04	-1.33	0.58	0.49	1.17	corr = 0.87, p < 0.001

Supplemental Figure S3. Soft clustering of gene expression in *Cuscuta campestris*. (A) Correlation matrix between cluster center values. Clusters are ordered after hierarchical clustering using the complete-linkage method. Rectangles delineate groups of similar clusters. (B) Cluster center values. For each cluster, a darker color indicates the trait (pattern of expression) that fits best, as determined by a point-biserial correlation test between centers and all possible combination of stages across host-free and host-induced systems (with 1 indicating expression in one or several stages and 0 indicating no expression in the others). Corresponding correlation indices and p-values are reported on the right side of the map. niS = non-infective stem, SWE = swelling stage, ATT = attaching stage, PEN = penetrating stage.

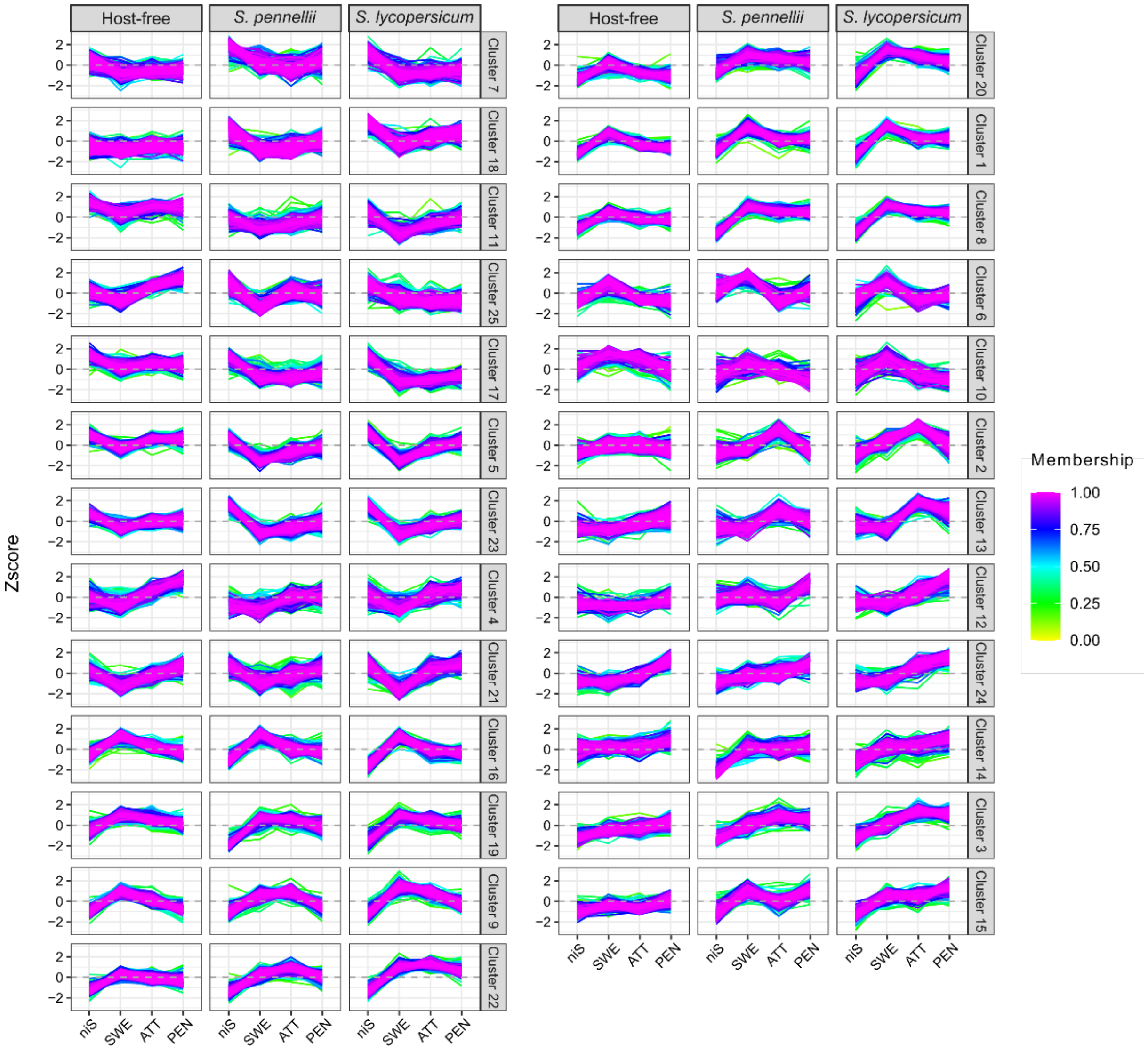

Supplemental Figure S4. Gene expression profiles (*Cuscuta campestris*) in soft clusters. Values are "Transcripts Per Kilobase Million" (TPM) values transformed into z-scores. A z-score value is positive (negative) if gene expression in a sample is larger (smaller) than the mean expression for that gene. Colors indicate the membership value (the higher the value, the better a gene fits into a cluster). niS = non-infective stem, SWE = swelling stage, ATT = attaching stage, PEN = penetrating stage.

A

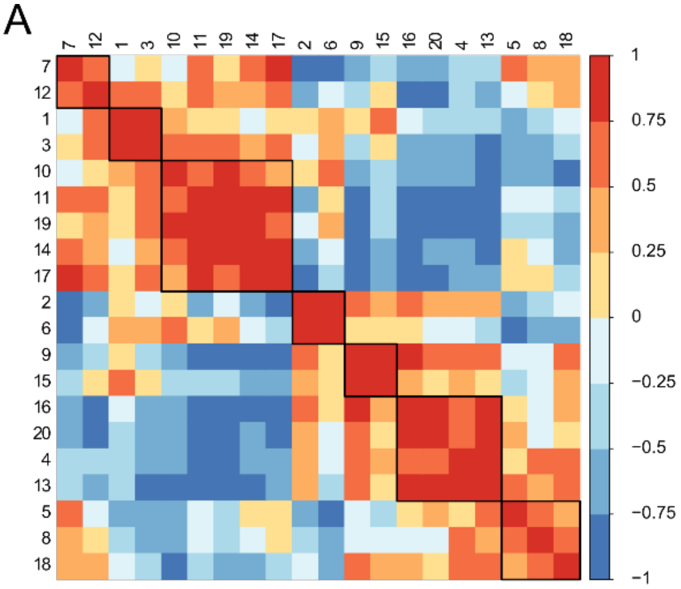

B

	S. pennellii				*S. lycopersicum*				
	niS	SWE	ATT	PEN	niS	SWE	ATT	PEN	
Cluster 7	0.69	0.93	0.77	0.83	-0.83	-0.87	-0.81	-0.71	corr = 0.99, p < 0.001
Cluster 12	0.34	0.58	0.68	0.63	-0.56	0.54	-0.95	-1.27	corr = 0.96, p < 0.001
Cluster 1	-0.06	-0.81	0.95	-0.19	-0.23	1.52	-0.44	-0.74	corr = 0.93, p = 0.001
Cluster 3	0.76	-0.66	0.85	-0.25	0.23	1.05	-1.03	-0.94	corr = 0.92, p = 0.001
Cluster 10	0.78	-0.38	-0.15	-0.61	1.58	0.40	-1.02	-0.61	corr = 0.88, p = 0.004
Cluster 11	1.07	0.36	0.47	0.12	0.80	-0.10	-1.48	-1.24	corr = 0.92, p = 0.001
Cluster 19	1.07	0.02	0.14	-0.22	1.30	0.08	-1.37	-1.01	corr = 0.81, p = 0.015
Cluster 14	0.75	0.20	0.57	0.19	1.02	-0.81	-1.56	-0.35	corr = 0.87, p = 0.005
Cluster 17	1.04	0.62	0.80	0.38	0.18	-0.63	-1.37	-1.02	corr = 0.93, p = 0.001
Cluster 2	-0.87	-0.75	-0.87	-0.71	0.77	1.15	0.68	0.59	corr = 0.98, p < 0.001
Cluster 6	-0.21	-0.72	-0.52	-0.75	1.33	1.17	-0.15	-0.15	corr = 0.95, p < 0.001
Cluster 9	-0.99	-0.58	-0.53	-0.10	-0.71	1.07	1.20	0.63	corr = 0.94, p = 0.001
Cluster 15	-0.74	-0.63	0.42	-0.10	-0.91	1.49	0.57	-0.11	corr = 0.85, p = 0.007
Cluster 16	-0.78	-0.64	-0.72	-0.41	-0.38	0.27	1.47	1.19	corr = 0.93, p = 0.001
Cluster 20	-0.54	-0.52	-0.36	-0.38	-0.40	-0.64	1.75	1.09	corr = 0.98, p < 0.001
Cluster 4	-1.22	0.40	-0.99	0.69	-0.64	0.34	0.70	0.71	corr = 0.97, p < 0.001
Cluster 13	-0.84	0.00	-0.66	0.47	-0.89	-0.39	1.27	1.05	corr = 0.91, p = 0.002
Cluster 5	0.05	0.52	0.08	0.63	-0.66	-1.48	0.08	0.78	corr = 0.88, p = 0.004
Cluster 8	-0.62	1.28	-0.63	1.15	-0.63	-0.39	-0.37	0.21	corr = 0.94, p = 0.001
Cluster 18	-0.76	0.52	-0.02	0.91	-1.42	0.28	0.48	0.01	corr = 0.89, p = 0.003

Supplemental Figure S5. Soft clustering of gene expression in tomato hosts. (A) Correlation matrix between cluster center values. Clusters are ordered after hierarchical clustering using the complete-linkage method. Rectangles delineate groups of similar clusters. (B) Cluster center values. For each cluster, a darker color indicates the trait (pattern of expression) that fits best, as determined by a point-biserial correlation test between centers and all possible combination of stages across the two pathosystems (with 1 indicating expression in one or several stages and 0 indicating no expression in the others). Corresponding correlation indices and p-values are reported on the right side of the map. niS = non-infective stem, SWE = swelling stage, ATT = attaching stage, PEN = penetrating stage.

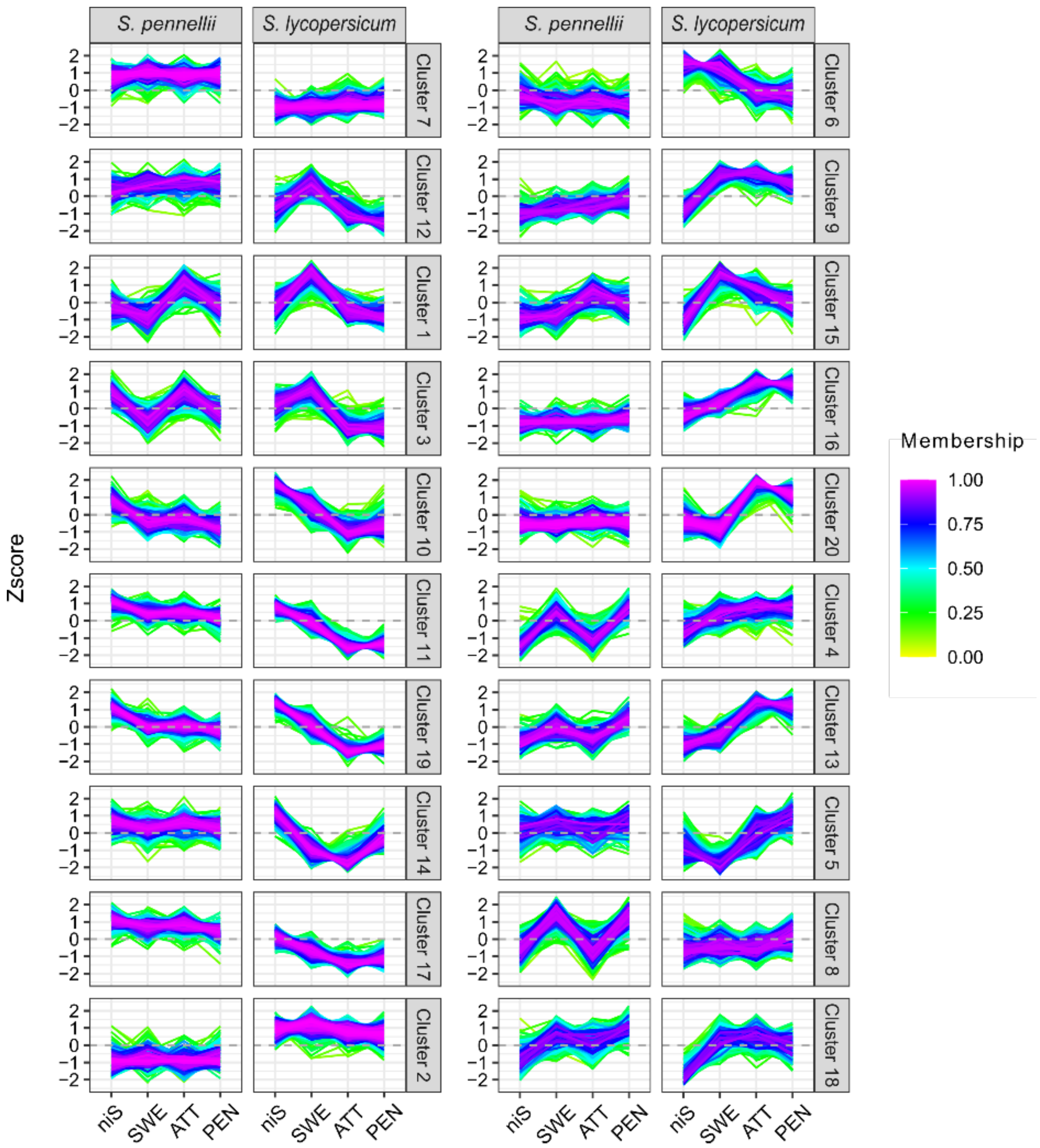

Supplemental Figure S6. Gene expression profiles (tomato hosts) in soft clusters. Values are "Transcripts Per Kilobase Million" (TPM) values transformed into z-scores. A z-score value is positive (negative) if gene expression in a sample is larger (smaller) than the mean expression for that gene. Colors indicate the membership value (with the higher the value, the better a gene fits into a cluster). niS = non-infective stem, SWE = swelling stage, ATT = attaching stage, PEN = penetrating stage.

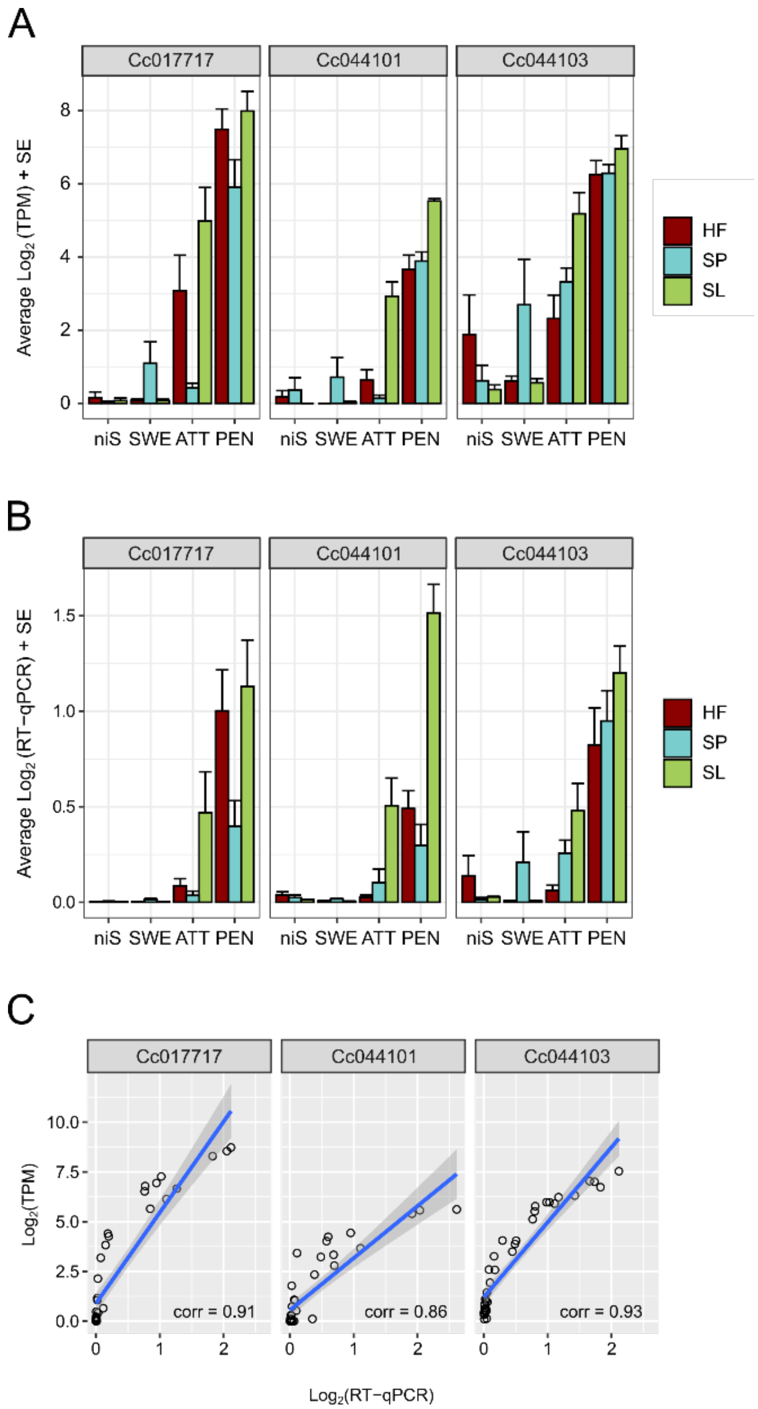

Supplemental Figure S7. Expression profile of selected endo-β-1,4-mannanases in *Cuscuta campestris*. (A) Average log-transformed "Transcripts Per Kilobase Million" (TPM) values in sequenced samples. Error bars indicate standard error (SE) of the mean (three biological replicates). (B) Gene expression as measured by reverse transcription (RT)-quantitative (q)PCR. Data were normalized against Cc006757 and Cc028808. Error bars indicate standard error of the mean (three biological replicates). (C) Pearson correlation coefficients (corr) with 95% confidence interval (shaded areas) between TPM values in sequenced samples and transcript abundance as measured by RT-qPCR. HF = host-free, SP = *Solanum. pennellii*, SL = *S. lycopersicum*, niS = non-infective stem, SWE = swelling stage, ATT = attaching stage, PEN = penetrating stage.

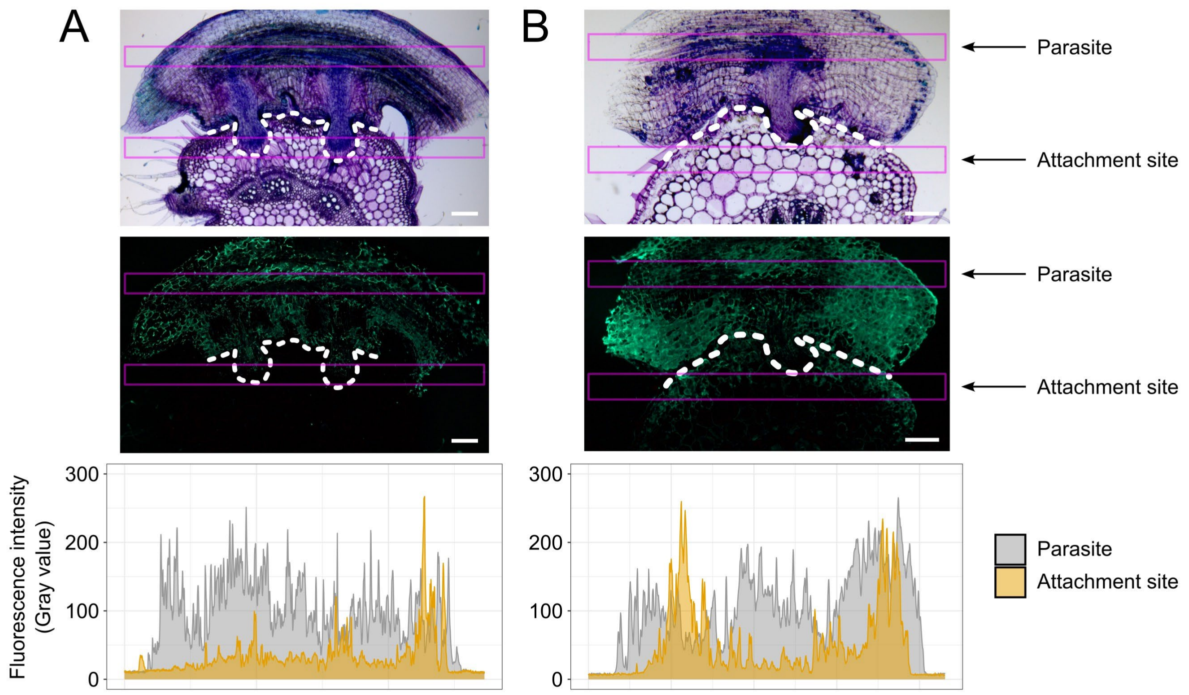

Supplemental Figure S8. Profiling of fluorescence intensity in tomato infection sites. (A) Distribution of mannan epitopes in a *C. campestris* infection site on a *S. pennellii* petiole using the LM21 antibody. The pictures are from Figures 7D and E and show a TBO-stained cross section and corresponding immunofluorescence. Dot lines delineate the interface at the contact region between parasite and host. Rectangles indicate the regions in the parasite stem and tomato petiole (attachment site) where intensity profiles were measured using Fiji v. 2.3.0 (Schindelin et al., 2012). The plot shows fluorescence intensity across the regions with parasite stem (grey) as background and attachment site (yellow) in the forefront. Intensity barely increases in the petiole under the attachment ring; the peaks here correspond to haustorial tissues crossing path. (B) Distribution of mannan epitopes in a *C. campestris* infection site on a *S. lycopersicum* petiole. The pictures are from Figures 7H and I. Profiling fluorescence intensity underneath the attachment ring here clearly identifies regions where mannan polymers accumulate in response to attempted parasitism. The decrease in intensity around the haustorium may indicate to mannan degradation by the parasite. Scale bars are 200 µm.

Supplemental Table S1. Quality filtering and trimming statistics.

System	Sample	Raw_pairs	Read_length	Q20	Q30	%GC	Clean_pairs	%Clean	Read_length	Q20	Q30	%GC
HF	niS1	15322713	101	99.85	98.08	47.09	14063741	91.78	85-101	100	99.96	47.02
HF	niS2	15443819	101	99.84	98.21	46.92	14212423	92.03	85-101	100	99.96	46.85
HF	niS3	15727264	101	99.85	98.08	47.29	14377358	91.42	85-101	100	99.96	47.21
HF	SWE1	15781639	101	99.85	98.23	47.48	14513192	91.96	85-101	100	99.97	47.42
HF	SWE2	15814546	101	99.86	98.29	47.10	14617190	92.43	85-101	100	99.97	47.03
HF	SWE3	15748623	101	99.85	98.21	46.80	14492184	92.02	85-101	100	99.97	46.76
HF	ATT1	15687672	101	99.85	98.13	47.44	14401468	91.80	85-101	100	99.96	47.37
HF	ATT2	15720768	101	99.86	98.18	47.28	14432949	91.81	85-101	100	99.97	47.21
HF	ATT3	15317696	101	99.85	98.24	47.29	14123864	92.21	85-101	100	99.97	47.23
HF	PEN1	15454773	101	99.87	98.31	47.65	14300205	92.53	85-101	100	99.97	47.59
HF	PEN2	15864650	101	99.86	98.26	47.43	14683339	92.55	85-101	100	99.97	47.39
HF	PEN3	15497676	101	99.84	98.11	47.35	14277601	92.13	85-101	100	99.96	47.29
SP	niS1	63932255	150	99.96	97.12	44.57	57738459	90.31	135-150	100	99.96	44.64
SP	niS2	65622418	150	99.96	97.12	44.46	59392568	90.51	135-150	100	99.96	44.48
SP	niS3	52032582	150	99.95	96.55	43.89	46457787	89.29	135-150	100	99.95	43.94
SP	SWE1	51246053	150	99.95	96.67	46.60	45487473	88.76	135-150	100	99.95	46.63
SP	SWE2	52972165	150	99.93	96.31	46.12	46937023	88.61	135-150	100	99.95	46.20
SP	SWE3	51374908	150	99.94	96.83	45.94	45955084	89.45	135-150	100	99.95	46.04
SP	ATT1	50433547	150	99.95	96.87	45.96	45230211	89.68	135-150	100	99.95	46.01
SP	ATT2	66382356	150	99.91	95.33	46.60	57993308	87.36	135-150	100	99.93	46.77
SP	ATT3	51193326	150	99.94	96.58	45.72	45445339	88.77	135-150	100	99.95	45.84
SP	PEN1	53111496	150	99.95	97.34	44.94	48672528	91.64	135-150	100	99.97	44.96
SP	PEN2	50663155	150	99.95	96.67	46.75	45336007	89.49	135-150	100	99.95	46.85
SP	PEN3	62146261	150	99.93	94.86	45.40	53974367	86.85	135-150	100	99.92	45.51

Supplemental Table S1 (Continued).

System	Sample	Raw_pairs	Read_length	Q20	Q30	%GC	Clean_pairs	%Clean	Read_length	Q20	Q30	%GC
SL	niS1	54765718	150	99.96	97.22	44.36	49620392	90.60	135-150	100	99.96	44.41
SL	niS2	54856570	150	99.96	97.06	43.79	49563799	90.35	135-150	100	99.95	43.85
SL	niS3	61183897	150	99.94	94.83	44.02	53265649	87.06	135-150	100	99.92	44.10
SL	SWE1	50266958	150	99.95	97.19	46.50	45557081	90.63	135-150	100	99.96	46.56
SL	SWE2	62114801	150	99.94	96.77	46.72	55777071	89.80	135-150	100	99.96	46.75
SL	SWE3	59145946	150	99.91	95.47	45.84	51725851	87.45	135-150	100	99.93	45.99
SL	ATT1	60663581	150	99.92	94.77	46.35	52627085	86.75	135-150	100	99.92	46.44
SL	ATT2	73924929	150	99.95	96.83	45.25	66424317	89.85	135-150	100	99.95	45.31
SL	ATT3	59052916	150	99.95	97.12	45.48	53300189	90.26	135-150	100	99.96	45.52
SL	PEN1	54362644	150	99.96	96.89	44.63	48854410	89.87	135-150	100	99.96	44.66
SL	PEN2	63501885	150	99.95	96.65	44.46	56868987	89.55	135-150	100	99.95	44.49
SL	PEN3	69994213	150	99.92	94.35	45.00	60440980	86.35	135-150	100	99.92	45.07

Supplemental Table S2. Mapping statistics.

System	Sample	Total _pairs	Uniquely _mapped	Multi- _mapped	Un- mapped	Assigned _to feature	Perc _assigned	Parasite	Percent _parasite	Host	Percent host
HF	niS1	14063741	12624533	1145507	293701	11687939	92.58	11687911	100.00	28	0.00
HF	niS2	14212423	12670237	1227681	314505	11620080	91.71	11620025	100.00	55	0.00
HF	niS3	14377358	12790100	1284632	302626	11738902	91.78	11738865	100.00	37	0.00
HF	SWE1	14513192	12859119	1340069	314004	12029664	93.55	12029640	100.00	24	0.00
HF	SWE2	14617190	13039551	1295663	281976	12086950	92.69	12086909	100.00	41	0.00
HF	SWE3	14492184	12938678	1281138	272368	12102782	93.54	12102761	100.00	21	0.00
HF	ATT1	14401468	12863693	1249862	287913	11973098	93.08	11973054	100.00	44	0.00
HF	ATT2	14432949	12830974	1304412	297563	11879506	92.58	11879476	100.00	30	0.00
HF	ATT3	14123864	12604293	1223184	296387	11670662	92.59	11670612	100.00	50	0.00
HF	PEN1	14300205	12799576	1223748	276881	11900303	92.97	11900282	100.00	21	0.00
HF	PEN2	14683339	13132626	1280775	269938	12138768	92.43	12138742	100.00	26	0.00
HF	PEN3	14277601	12813002	1201132	263467	11893965	92.83	11893929	100.00	36	0.00
SP	niS1	57738459	52273226	3052622	2412611	47905564	91.64	20897308	43.62	27008256	56.38
SP	niS2	59392568	54764100	2675080	1953388	50697575	92.57	19115606	37.71	31581969	62.29
SP	niS3	46457787	42644112	2023197	1790478	39783624	93.29	9892791	24.87	29890833	75.13
SP	SWE1	45487473	42662403	2064954	760116	40183162	94.19	33084200	82.33	7098962	17.67
SP	SWE2	46937023	43444535	2411355	1081133	40552273	93.34	31573921	77.86	8978352	22.14
SP	SWE3	45955084	42551679	2226743	1176662	39717173	93.34	31731405	79.89	7985768	20.11
SP	ATT1	45230211	42205985	2064439	959787	39275594	93.06	29752252	75.75	9523342	24.25
SP	ATT2	57993308	53435950	3190056	1367302	49538942	92.71	44890104	90.62	4648838	9.38
SP	ATT3	45445339	41302160	2612759	1530420	38225320	92.55	30158130	78.90	8067190	21.10
SP	PEN1	48672528	45364621	2044326	1263581	41965182	92.51	22633545	53.93	19331637	46.07
SP	PEN2	45336007	42601247	2002831	731929	39930348	93.73	34928312	87.47	5002036	12.53
SP	PEN3	53974367	49998813	2423521	1552033	46636407	93.28	31654612	67.88	14981795	32.12

Supplemental Table S2 (Continued).

System	Sample	Total pairs	Uniquely mapped	Multi-mapped	Un-mapped	Assigned to feature	Percent assigned	Parasite	Percent parasite	Host	Percent host
SL	niS1	49620392	46942366	1555322	1122704	43419699	92.50	14088248	32.45	29331451	67.55
SL	niS2	49563799	46899872	1537927	1126000	43210810	92.13	8885663	20.56	34325147	79.44
SL	niS3	53265649	50848178	1526854	890617	46962014	92.36	10638545	22.65	36323469	77.35
SL	SWE1	45557081	42701997	2233111	621973	39710621	92.99	35424131	89.21	4286490	10.79
SL	SWE2	55777071	52487538	2582637	706896	48792726	92.96	45120441	92.47	3672285	7.53
SL	SWE3	51725851	48288704	2571336	865811	44770246	92.71	40140984	89.66	4629262	10.34
SL	ATT1	52627085	49574643	2249136	803306	46047754	92.89	37119738	80.61	8928016	19.39
SL	ATT2	66424317	62420797	2883887	1119633	57218004	91.66	41012662	71.68	16205342	28.32
SL	ATT3	53300189	49666319	2628507	1005363	45518952	91.65	28863308	63.41	16655644	36.59
SL	PEN1	48854410	46634857	1556871	662682	43159921	92.55	16700014	38.69	26459907	61.31
SL	PEN2	56868987	54406049	1715670	747268	50168227	92.21	19231877	38.33	30936350	61.67
SL	PEN3	60440980	57237375	2250999	952606	53220535	92.98	27062794	50.85	26157741	49.15

Supplemental Table S3. Primer sequence pairs for selected endo-β-1,4-mannanases with their amplicon sizes.

Accession	Forward (5'-3')	Reverse (5'-3')	Amplicon (pb)
Cc017717	GGCAACAAGACGAGTGAGGA	CGTGGTGTTGGAAATGGCAG	198
Cc044101	ACTGTTGCTAGGACTTGGGC	ACGTAATCCAGCGCCTGAAA	101
Cc044103	GCGAGGAACCACGGTCTAAA	CGACATAATCCAGCGCCTCT	127

Gene-specific forward and reverse primer pairs were designed using Primer3 (v. 2.4.0) (Untergasser et al., 2012). Target specificity was verified using the blastn-short tool from the NCBI BLAST+ suite (v. 2.6.0) (Altschul et al., 1990). Both software were used as standalone to enable batch computing.

REFERENCES

Altschul SF, Gish W, Miller W, Myers EW, Lipman DJ (1990) Basic local alignment search tool. J Mol Biol **215**: 403–10

Bawin T, Bruckmüller J, Olsen S, Krause K (2022) A host-free transcriptome for haustoriogenesis in Cuscuta campestris: Signature gene expression identifies markers of successive development stages. Physiol Plant **174**: e13628

Schindelin J, Arganda-Carreras I, Frise E, Kaynig V, Longair M, Pietzsch T, Preibisch S, Rueden C, Saalfeld S, Schmid B, et al (2012) Fiji: an open-source platform for biological-image analysis. Nat Methods **9**: 676–82

Untergasser A, Cutcutache I, Koressaar T, Ye J, Faircloth BC, Remm M, Rozen SG (2012) Primer3--new capabilities and interfaces. Nucleic Acids Res **40**: e115
